# Characterization of the Estrogen Response Helps to Predict Prognosis and Identify Potential Therapeutic Targets in Cholangiocarcinoma

**DOI:** 10.3389/fonc.2022.870840

**Published:** 2022-05-19

**Authors:** Chenglin Lu, Ji Miao, Minhuan Li, Qisi Zheng, Feng Xu, Yiming Pan, Yizhou Wang, Zhi Yang, Xuefeng Xia, Hao Zhu, Jie Chen, Shanhua Bao

**Affiliations:** ^1^Department of General Surgery, Nanjing Drum Tower Hospital, The Affiliated Hospital of Nanjing University Medical School, Nanjing, China; ^2^Department of Andrology, Nanjing Drum Tower Hospital, The Affiliated Hospital of Nanjing University Medical School, Nanjing, China; ^3^Department of Laboratory Medicine, Nanjing Drum Tower Hospital, The Affiliated Hospital of Nanjing University Medical School, Nanjing, China; ^4^Department of Anesthesiology, Nanjing Drum Tower Hospital, The Affiliated Hospital of Nanjing University Medical School, Nanjing, China; ^5^Department of Gastroenterology, Nanjing Drum Tower Hospital, The Affiliated Hospital of Nanjing University Medical School, Nanjing, China; ^6^Department of General Surgery, Changshu NO.1 People’s Hospital, The Affiliated Hospital of Soochow University, Changshu, China

**Keywords:** Cholangiocarcinoma, estrogen response, estrogen metabolism, complement activation, chemotherapeutic targets

## Abstract

Cholangiocarcinoma (CCA) is an aggressive malignancy originating from the epithelium of the bile duct. The prognosis of patients is poor regardless of radical resection and chemoradiotherapy. The current classification and prognostic model of CCA are unable to satisfy the requirements for predicting the clinical outcome and exploring therapeutic targets. Estrogen signaling is involved in diverse cancer types, and it has long been established that CCA could be regulated by estrogen. In our study, estrogen response was identified to be significantly and stably correlated with poor prognosis in CCA. Employing several algorithms, CCA was classified into ES cluster A and B. ES cluster B was mainly composed of patients with fluke infection and overlapped with CCA cluster 1/2, and ES cluster A was mainly composed of patients without fluke infection and overlapped with CCA cluster 3/4. COMT and HSD17B1 were identified to be responsible for the differential estrogen response between ES clusters A and B, and the estrogen response may be correlated with the differentiation and cancer stemness of CCA at the single-cell level. Complement activation and the expression of C3 and C5, which are mainly expressed by CCA cells, were significantly downregulated in ES cluster B. An estrogen response risk score (ESRS) model was constructed to predict the prognosis of CCA, followed by a nomogram integrating ESRS and clinical features. Finally, altered pathways, applicable drugs and sensitivity to chemical drugs were analyzed specific to the estrogen response. In summary, our results provide insights into the role of the estrogen response in CCA progression as well as applicable drugs and potential therapeutic targets in estrogen metabolism, the complement system and ESRS-related pathways.

## Introduction

Cholangiocarcinoma (CCA) is one of the most malignant cancers derived from cholangiocytes of the bile duct (1, 2). The epidemiology of CCA varies around the world. Fluke infection is the leading cause of CCA in some regions, such as Thailand (3). In the Western world, primary sclerosing cholangitis (PSC) is the only identifiable risk factor (4). CCA can be subdivided into intrahepatic (iCCA), perihilar (pCCA), and distal CCA (dCCA) according to anatomic location and differential treatment (5). Surgery is the preferred treatment for early resectable CCA. Most patients with CCA are diagnosed at late stages due to a lack of obvious symptoms (~70%), and only 25% of them are recommended for surgery (6, 7). Unfortunately, patients with unresectable CCA share a median overall survival time of less than 1 year (8). For patients after radical resection of CCA, the 5-year overall survival is lower than 30% (9). In addition, current radiotherapy and chemotherapy show limited effects on patients with CCA (8). Existing CCA classification is mainly based on anatomic location or pathologic features. Until recently, the International Cancer Genome Consortium classified CCA into CCA clusters 1-4 based on integrated analysis of genomic, epigenomic, and transcriptomic information of nearly 500 CCAs from 10 countries, which provided insights into the mechanisms of tumorigenesis of CCA as well as potential therapeutic targets (10). Further studies in the classification and characterization of CCA are necessary and instrumental in breaking the predicament of the poor prognosis of patients with CCA.

Beyond physiological functions in regulating the menstrual cycle, reproduction, bone density, brain function and cholesterol mobilization, estrogen is associated with the development and progression of various types of cancer, such as cancers in the breast, ovary, endometrium, colon, prostate and lung (11). The role of estrogen also varies among different cancer types, which are mediated by the activation of the different estrogen receptor (ER) subtypes: ERα and ERβ. Generally, ERα is involved in proliferation, inflammation and tumorigenesis and is responsible for the adverse effect of estrogen. ERβ is involved in the suppression of proliferation, differentiation and apoptosis and is responsible for the beneficial effect of estrogen (12). Estrogen also plays an important role in the tumor immune microenvironment (TIME). For instance, estrogen may promote the accumulation of myeloid-derived suppressor cells (MDSCs) and impair the antitumor effect of CD8^+^ T cells (13). In addition, the aberrant estrogen effect could also arise from the fluctuation of estrogen concentration by change of endogenous or exogenous estrogen or the disorder of estrogen metabolism. For example, under chronic inflammatory conditions, steroid sulfatase (STS), which can catalyze inactive estrogen sulfates to active estrogen, is upregulated, thus increasing the concentration of active estrogen in chronic liver disease (12).

Both ERα and ERβ are expressed in cholangiocytes (14, 15). Substantial evidence shows that estrogen promotes carcinogenesis and the development of CCA, and selective modulation of estrogen receptors could inhibit the growth of CCA (16–19). In addition, a meta-analysis enrolling 1,107,498 women of 12 North American-based cohort studies suggested that long-term use of oral contraceptives with only estrogen may be associated with an increased iCCA risk (20).

Until recently, however, the whole picture of the complexity of the estrogen response in CCA has remained obscure and largely incomplete. To what extent the estrogen response could influence CCA progression has not been determined. With the development of high-throughput sequencing and bioinformatic technologies, we are equipped to evaluate the level of estrogen response of CCA samples and even single cells. In this study, we systematically characterized the estrogen response in CCA. Furthermore, we will explore the mechanism of the differential estrogen response, the role of the estrogen response in the TIME and potential targets specific to the estrogen response (**Supplementary Figure 1**).

## Materials and Methods

### Data Gathering and Preprocessing

In our investigation, four microarray datasets from the GEO database (https://www.ncbi.nlm.nih.gov/gds/), including GSE26566, GSE33327, GSE76297 and GSE89749, a microarray dataset from the ArrayExpress database (https://www.ebi.ac.uk/arrayexpress/) named E-MTAB-6389, and an RNA-sequencing dataset from The Cancer Genome Atlas (TCGA, https://portal.gdc.cancer.gov/) named TCGA-CHOL, were downloaded. In brief, primary microarray data sets were preprocessed with background adjustment and normalization employing the R package for microarray data processing (21). Probe IDs or ensembl gene IDs were transferred into gene symbols according to the corresponding annotation file, and the median value was selected to represent the expression value for gene symbols with multiple probes. All the expression data enrolled in this study were log2 transformed. Only the survival information of the GSE89749 cohort, E-MTAB-6389 cohort and TCGA-CHOL cohort was publicly accessible. In addition, a single-cell sequencing dataset for 5 iCCA samples was also retrieved from the GEO database, named GSE138709 (22). Basic information on the datasets used in this study is summarized in **Supplementary Table S1**.

### Weighted Gene Coexpression Network Analysis

All microarray datasets of CCA samples, including GSE26566 (n = 104), GSE33327 (n = 149), GSE76297 (n = 91), GSE89749 (n = 118) and E-MTAB-6389 (n = 78), were merged and followed with bath correction. The expression of the top 30% genes with the highest variance in the merged dataset was performed with WGCNA utilizing the R package WGCNA 1.70.3. First, the optimal soft thresholding power β of five was selected to construct a scale-free network topology. Then, a topological overlap matrix (TOM) was generated out of the adjacency matrix to calculate the corresponding dissimilarity. Genes were hierarchically clustered to produce a dendrogram that was divided into different gene modules using the dynamic tree cut method. The eigengene was calculated to represent each module, and the most relevant gene module for specific traits was evaluated by Spearman correlation analysis.

### Gene Set Enrichment Analysis and Gene Set Variation Analysis

The hallmark gene sets, biological processes and canonical pathways were downloaded from the Molecular Signatures Database (MSigDB, http://www.gsea-msigdb.org/gsea/index.jsp). GSEA was performed to demonstrate the altered biological processes or canonical pathways between different patient groups. The R package fgsea 1.18.0 was employed to carry out the GSEA and plot the results. GSVA was performed to evaluate the relative level of specific biological processes for a single sample. The R package GSVA 1.40.1 was employed to evaluate the GSVA score for each sample with the “ssgsea” method.

### Single Cell Data Processing

For the analysis of single-cell sequencing data, the R package Seurat 4.0.5 was used to process the raw data of GSE138709 (23). Only CCA samples of GSE138709 were loaded followed by filtration with the criteria of >20% mitochondria-related genes and more than 1,000 genes.

Expressed per cell. A total of 17,090 cells were selected for subsequent analysis. After data normalization, identification of variable features and principal component analysis (PCA) dimensional reduction, the dataset was batch corrected by the “RunHarmony” function of the R package 0.1.0 All the cells were clustered into 16 cell populations using the “FindClusters” function of Seurat (resolution = 0.5). TSNE dimensionality reduction was performed to generate the TSNE plot. The annotation of the cell clusters was fulfilled by the R package singleR 1.6.1 and adjusted by signatures from the original publication (22). EPCAM, KRT7, KRT19, ANXA4 and TM4SF4 were chosen as markers for CCA cells. The AUCell score of specific gene sets for single cells was calculated using the R package irGSEA 1.0.0. Monocle 2.30.0 was employed to perform the cell trajectory analysis.

### Survival Analyses

The survival curves were plotted with the R package survminer 0.4.9. In brief, the Kaplan–Meier method was employed for the drawing, and the log-rank test was used for evaluation of differences in survival. The best cutoff value of parameters was calculated by the ‘surv_cutpoint’ function. The R package survivalROC 1.0.3 was used to plot the time-dependent receiver operating characteristic curve (ROC) for prognostic factors.

### Establishment of the Estrogen Response-Related Score

First, the GSE89749 cohort and E-MTAB-6389 cohort were merged and followed with batch correction. Samples without valid survival information were removed. Then, the merged dataset was randomly divided into a training cohort (n = 128) and a testing cohort (n = 55) at a ratio of 7:3 *via* the “sample” function in R. Subsequently, we performed univariate Cox regression for genes in the gene set “Estrogen response late” using the “coxph” function of the R package survival 3.2.13, and 65 genes with a p value less than 0.1 were included in the least absolute shrinkage and selection operator (LASSO) regression by the R package glmnet 4.1.2. In our model, 11 genes maintained their Cox coefficients under a tuned penalty parameter (λ). The ESRS was established based on multivariate regression of the selected genes. The stability of the model was further tested in the testing cohort. We also integrated the ESRS with the clinical information of CCA patients to construct a nomogram with the R package regplot 1.1. A calibration curve was plotted to check the veracity of the nomogram using the R package rms 6.2.0.

### Additional Bioinformatics and Statistical Analyses

The differential expression of genes between patient groups was calculated using the R package limma 3.48.3. Correlation analysis was performed using the R package Hmisc 4.6.0. The immune infiltration of 22 types of immune cells was estimated by the R package Cibersort at 1,000 permutations. Unsupervised clustering analysis was carried out to define the optimal clustering of CCA samples based on the expression of genes related to the estrogen response by the R package ConsensusClusterPlus 1.56.0 at 1,000 permutations, and the R package Rtsne 0.15 was employed to visualize the clustering. The protein–protein interaction (PPI) networks were constructed by the STRING database (https://cn.string-db.org/) and visualized by Cytoscape 3.8.2 software. The network of biological processes based on genes in estrogen response-related genes was generated by ClueGO, a plug-in of Cytoscape. A connectivity map (CMap) was employed to identify potential targets and drugs for the ESRS_high group. The IC50 of each chemical compound of each cancer cell line along with the RNA sequencing data of all the cancer cell lines were downloaded from the Genomics of Drug Sensitivity in Cancer database (GDSC, https://www.cancerrxgene.org/) To which drugs the CCA patients with high estrogen response are sensitive or resistant is evaluated by Spearman correlation analysis between the GSVA score of “Estrogen response late” and IC50 of the drugs in all cancer cell lines.

For comparison of two groups, Student’s t test and Wilcoxon rank-sum tests were employed to estimate the statistical significance of normally distributed and nonnormally distributed variables, respectively. For comparisons of more than two groups, Kruskal–Wallis tests were used. Differences with p < 0.05 were considered statistically significant. Statistical p value (*, P < 0.05; **, P < 0.01; ***, P < 0.001; ****, P<0.0001; ns, not significant).

## Results

### Identification of an Estrogen Response-Related Gene Set

**Figure 1:**
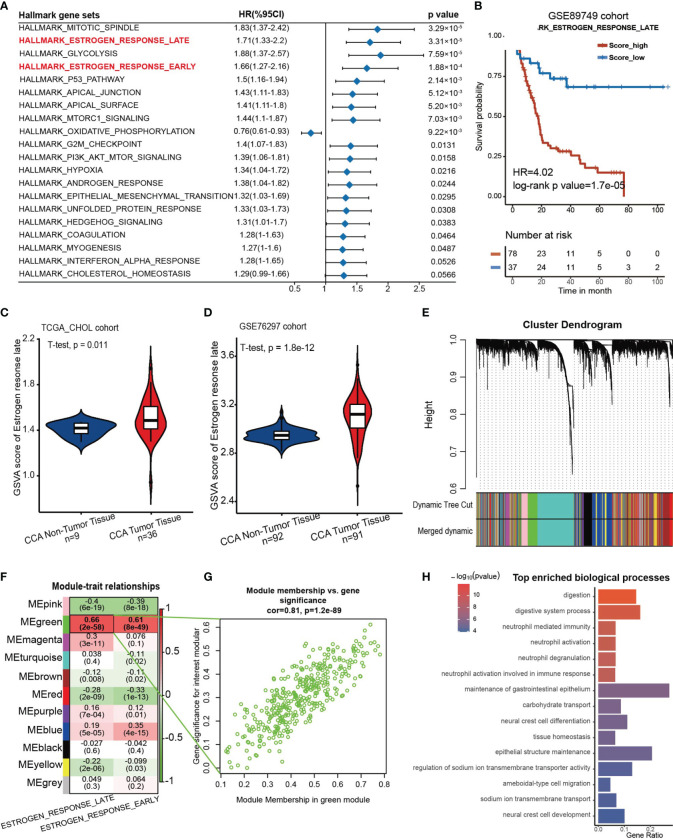
An estrogen response-related gene set was identified. **(A)** Forest plot showing the prognostic value of the GSVA score of the top 20 hallmark gene sets in the GSE89749 cohort. The horizontal coordinates represent the univariate regression coefficient of the normalized GSVA score of each hallmark of cancer, and the horizontal line represents the 95% confidence interval of the univariate regression coefficient. **(B)** Kaplan–Meier curves for overall survival of the patients with high or low GSVA scores of “Estrogen response late” in the GSE89749 cohort. Score_low, n = 37; Score_high, n = 78. HR=4.02, Log-rank test, p value = 1.7×10^-5^. **(C, D)** Violin plots showing the GSVA score of “ HALLLMARK_ESTROGEN_RESPONSE_LATE” in CCA tumoral and non-tumoral tissues in TCGA_CHOL cohort and GSE76297 cohort. **(E)** Weighted gene coexpression network analysis (WGCNA) was performed with an expression matrix of 462 samples in the meta cohort to construct a scale-free coexpression network. The cluster dendrogram shows the clustering of genes into gene modules. **(F)** Eleven gene modules were generated, and a green module exhibited the highest correlation with the GSVA score of “Estrogen response late” (Spearman correlation test, r= 0.9, P = 2e-58) and was considered an “ES-specific module”. **(G)** Scatter plot showing the Spearman correlation coefficient between the expression of genes in the green module and the eigengene of the green module or the GSVA score of “Estrogen response late”. **(H)** Bar plot showing the top 15 enriched biological processes in the green module.

First, the GSE89749 cohort containing 118 CCA samples was chosen as the discovery cohort due to the appropriate sample size and clinical information. Then, we scored the active level of all the hallmark gene sets utilizing the GSVA algorithm, and the result was further subjected to univariate Cox regression. It showed that “Estrogen response late” along with “Estrogen response early” were significantly correlated with poor prognosis (**Figure 1A**). These two gene sets were curated by MSigDB according to transcriptomic alterations in early (within 6 hours) or late (over 12 hours) stage of cellular response to estrogen. The Kaplan–Meier curves for the GSVA score of these two gene sets in the GSE89749 cohort, E-MTAB-6389 cohort and TCGA-CHOL cohort were plotted, and only “Estrogen response late” showed stable predictive capability for prognosis (**Figure 1B**, HR=4.02, log-rank test p value= 1.7×10^-5^; **Supplementary Figures 2A–E**). Through dimensionality reduction of 200 genes in “Estrogen response late” for 32 types of cancer in TCGA cohort, we found that the response to estrogen differs in almost all cancer types (**Supplementary Figure 2F**). Additionally, the prognostic value of the GSVA score of “Estrogen response late” varies among different solid cancer types, and the hazard ratio of which in CCA was highest of all the solid cancer types. Meanwhile, we investigated the difference in estrogen response score between non-tumoral and tumoral tissues in those cohorts with considerable sample size of normal bile duct tissues. Surprisingly, the estrogen response was stably upregulated in CCA tumor tissues, which further indicates the role of estrogen response in CCA progression (**Figures 1C, D**)

Subsequently, a scale-free coexpression network was established from the expression matrix of the GSE89749 cohort with the WGCNA algorithm. Genes were divided into 11 gene modules according to a similar expression pattern, and the green module was significantly correlated with the GSVA score of “Estrogen response late” (r=0.66, p=2×10^-58^) and “Estrogen response early” (r=0.61, p=8×10^-49^) (**Figures 1E** **–G**). Therefore, genes in the green module were identified as estrogen response-related genes (ESRGs). To determine the molecular function of ESRGs, gene enrichment analysis was performed and the results demonstrated that digestion, neutrophil activation and epithelial cell differentiation were among the top enriched biological processes (**Figure 1H**).

### Clinical and Transcriptomic Characteristics of Estrogen Response in the GSE89749 Cohort

**Figure 2:**
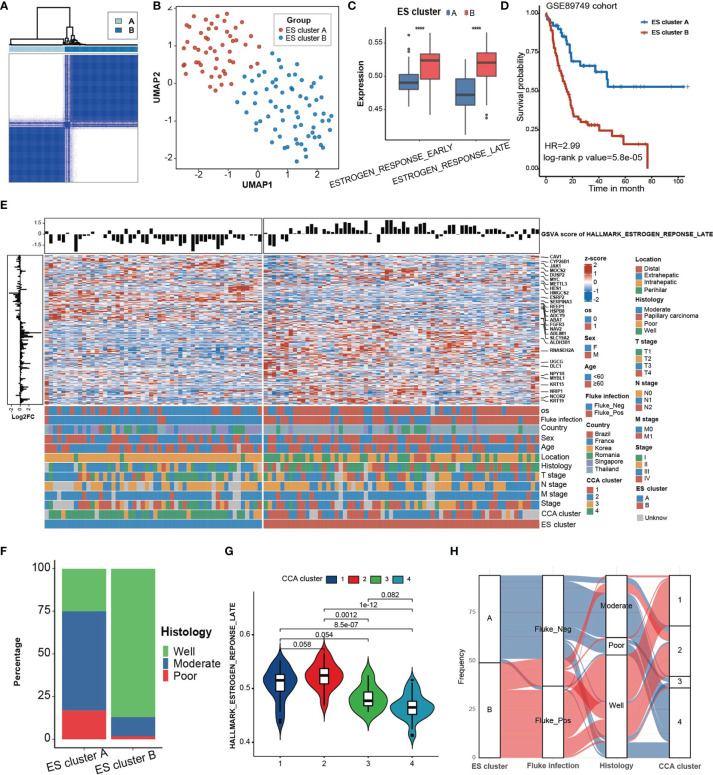
Clinical characteristics of ES clusters in the GSE89749 cohort. **(A)** Consensus matrixes of the GSE89749 cohort based on the expression of genes in the green module. K=2 was chosen as optimal clustering by delta area. **(B)** A UMAP plot of 118 patients by dimensionality reduction of genes in the blue module showing 2 distinct ES clusters. **(C)** ES cluster B exhibited significantly higher GSVA scores for “estrogen response late” and “estrogen response early” than ES cluster **(A, D)** Kaplan–Meier curves for overall survival of the patients in ES cluster A and ES cluster B in the GSE89749 cohort. ES cluster A, n = 51; Score_high, n = 64. HR = 2.99, Log-rank test, p value = 5.8×10^-5^. **(E)** Heatmap showing the expression of 198 genes involved in “estrogen response late” in 118 CCA patients in the GSE89749 cohort. Clinical information, including overall survival status, sex, age, fluke infection status, country, CCA clusters, location, histology, T stage, N stage, M stage, clinical stage, and ES clusters, is shown as patient annotation. The GSVA score of “Estrogen response early” of each patient was annotated on the top of the heatmap. The log2FoldChange of each gene between ES cluster A and ES cluster B is annotated on the left of the heatmap. **(F)** Stacked bar chart showing the proportion of different histological classifications in the ES cluster A and B groups in the GSE89749 cohort. **(G)** Violin plot showing the GSVA score of “Estrogen response early” in four subtypes of CCA clusters. **(H)** Alluvial diagram showing the relation among different ES clusters, fluke infection status, histology and CCA clusters. Statistical p-value ( ****,P < 0.0001).

Then, to investigate the clinical features of estrogen response, we attempted to cluster the CCA samples into different clusters on the basis of the expression of ESRGs in 118 CCA samples in the GSE89749 cohort. These samples were divided into two clusters (ES cluster A, n = 52; ES cluster B, n = 66) according to the optimal clustering chosen by delta area (**Figure 2A**, **Supplementary Figure 3A**). The UMAP plot based on dimensionality reduction of ESRGs indicated that these two clusters exhibited great transcriptome heterogeneity (**Figure 2B**). These two clusters showed significant differences in response to estrogen (**Figure 2C**) and overall survival probability (**Figure 2D**, HR = 2.99, log-rank p value = 5.8 ×10^-5^). Furthermore, the expression of genes in “Estrogen response late” also differed between the two clusters (**Figure 2E**).

The different clinical classification constitutions of the two ES clusters and the GSVA score of “Estrogen response late” in different clinical classifications were analysed (**Figure 2E**, **Supplementary Figure 3B**, **Supplementary Table S2**). ES cluster B contained the majority of people with fluke infection. Fluke infection is supposed to be a major cause of CCA in some regions, such as Thailand (3). Fluke infection could cause mechanical damage to cholangiocytes as well as chronic inflammation (24). Abnormal estrogen metabolism has long been detected in chronic liver disease (25). It has been reported that estrogen can act as an anti-inflammatory hormone and that STS chronic inflammation induced by chronic inflammation can increase the level of estrogen, which in turn alleviates the inflammatory response (26). Therefore, we hypothesized that the chronic inflammation caused by fluke infection may be responsible for the higher estrogen response in ES cluster B. In addition, ES cluster B was mainly composed of eCCA, which is mostly caused by fluke infection, and well-differentiated CCA (**Figure 2F**). The GSE89749 cohort was classified into 4 distinct CCA subtypes based on multiomics data (10). Our results indicated that CCA cluster 1 and 2 had higher GSVA scores for “Estrogen response late” than CCA cluster 3 and 4 (**Figure 2G**). Meanwhile, ES cluster A was mainly composed of CCA clusters 3 and 4, and ES cluster B was mainly composed of CCA clusters 1 and 2 (**Figure 2H**). As reported, CCA cluster 1/2 was enriched in ERBB2 amplifications and TP53 mutations. In addition, CCA cluster 3/4 are characterized by high copy number alterations and expression of PD-1/PD-L2 or epigenetic changes (IDH1/2, BAP1) along with FGFR/PRKA-related gene rearrangements. The overlaps of ES clusters and CCA subtypes indicated the potential of ES clusters in selecting personalized treatment, for example, promising IDH inhibitors (ClinicalTrials.gov identifier: NCT02073994). In addition, CCA had also been classified into “Inflammation” and “Proliferation” subtypes based on multiomics data (27). The inflammation-related class shows enrichment of inflammation and cytokine pathway, like overexpression of IL-6, IL-10, IL-17, and STAT3 constitutive activation, while the proliferation-related class shows more aggressive behavior, reflected by earlier recurrence and an enrichment of several oncogenic pathways, such as RTK signaling and angiogenesis-related pathways, and gene signatures of poor prognosis. Our results showed that the proliferation-related class showed significantly higher estrogen response than that in inflammation-related class (**Supplementary Figure 3C**).

We also analyzed the difference in biological processes between ES cluster A and ES cluster B (**Supplementary Figure 3D**). O-GlcNAcylation and glycosylation were significantly enriched in ES cluster B, which have been shown to mediate invasiveness and metastasis of CCA (28). The activation of complement was significantly suppressed in ES cluster B, indicating that the humoral immune response against CCA was compromised. Considering that a few metabolism-related processes were enriched, we analyzed several metabolism-specific gene sets between ES cluster A and B, including “Amino acid”, “Carbohydrate”, “Energy”, “Lipid”, “Nucleotide”, “TCA cycle”, and “Vitamin cofactor” (29). Among them, “TCA cycle” and “amino acid” were significantly downregulated in ES cluster B, and no difference was detected in other gene sets, indicating a role of the estrogen response in CCA metabolism (**Supplementary Figures S3E, F**).

### Potential Cause and Spatiotemporal Specificity of Differential Estrogen Response

**Figure 3:**
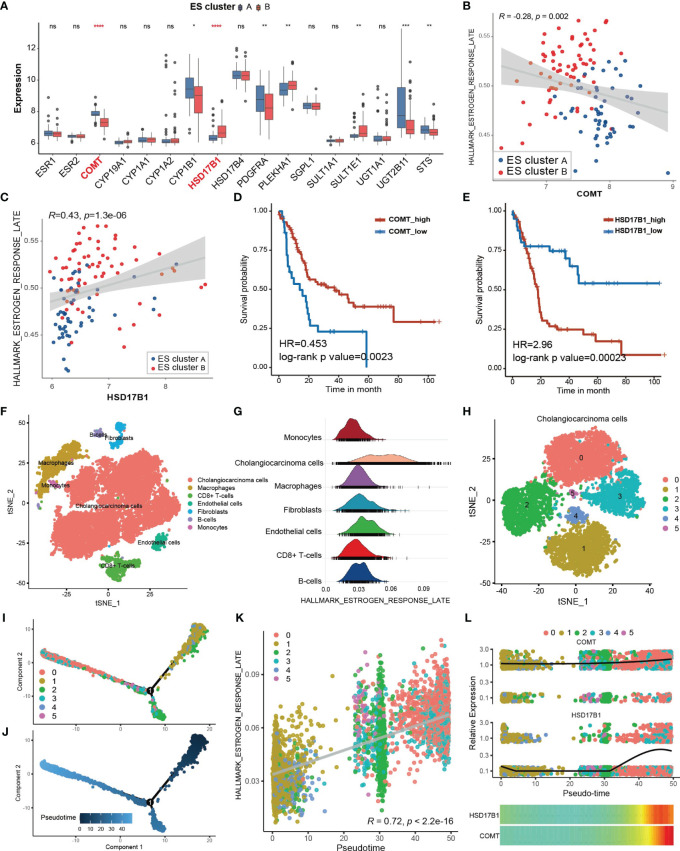
Potential cause and spatiotemporal specificity of the differential estrogen response. **(A)** Differential expression of estrogen receptors and estrogen metabolism-related genes. The top and bottom of the boxes represent the interquartile range of the values. The thick lines in the middle of the boxes represent the median values. The black dots show the outliers. The significant differences among different ECM clusters were evaluated using Student’s t test. **(B, C)** Scatter plot depicting the correlation between the GSVA score of “Estrogen response late” and the expression of COMT (Spearman correlation test, R = -0.28, p value =0.002) or HSD17B1 (Spearman correlation test, R = 0.43, p value =1.3×10^-6^). The color of the dots represents the ES clusters annotated by the legend. **(D, E)** Kaplan–Meier curves for overall survival of the patients with low or high expression of COMT or HSD17B1 in the GSE89749 cohort. **(F)** Single cell clusters for tumor tissues from five CCA samples. **(G)** Ridge plot showing the AUCell score of “Estrogen response late” in different cell types. **(H)** CCA cells were further divided into six subgroups exhibited by the TSNE plot. **(I, J)** Cell trajectory plot of cholangiocarcinoma cells annotated by subgroups of CCA cells and pseudotime. **(K)** Scatter plot showing the correlation between the AUCell score of “Estrogen response late” and the pseudotime of CCA cells. Spearman correlation test, r = 0.72, p <2.2×10^-16^. **(L)** The combined scatter plot and heatmap plot exhibiting the change in expression of COMT and HSD17B1 following pseudotime in CCA cells. Statistical p-value (*,P < 0.05; **,P < 0.01; ***,P < 0.001, ****,P < 0.0001). ns, not significant.

To explore the potential mechanism of the differential estrogen response in ES cluster A and B, we compared the expression of estrogen receptors and genes related to estrogen metabolism (**Figure 3A**). There was no difference in the expression of ESR1 (estrogen receptor alpha) and ESR2 (estrogen receptor beta) between the two ES clusters, while genes related to estrogen metabolism differed, especially catechol-O-methyltransferase (COMT) and hydroxysteroid (17beta) dehydrogenase 1 (HSD17B1). COMT could catalyze O-methylation of catechol estrogens and significantly compromise its estrogen receptor binding affinities (30). HSD17B1 efficiently catalyzes the conversion from estrone (E1) to the highly active estrogen estradiol (E2) (31). Coincidentally, COMT was significantly downregulated in ES cluster B, and HSD17B1 was significantly upregulated in ES cluster B. Then, we performed Spearman correlation analysis between the GSVA score of “Estrogen response late” and the expression of COMT and HSD17B1. As expected, the expression of COMT negatively correlated with the estrogen response (**Figure 3B**, Spearman correlation test, r = -0.28, p=0.002), and the expression of HSD17B1 positively correlated with the estrogen response (**Figure 3C**, Spearman correlation test, r = 0.43, p = 1.3×10^-6^). Furthermore, the expression of COMT was a favorable factor for CCA patients (**Figure 3D**, HR = 0.453, log-rank p value = 0.0023), and HSD17B1 was a risk factor for CCA patients (**Figure 3E**, HR = 0.453, log-rank p value = 0.00023).

Subsequently, we attempted to investigate the heterogeneity of the estrogen response within CCA tissues at the single-cell level. A total of 17,090 qualified single cells from cancer tissues of 5 CCA samples in the GSE138709 cohort were clustered after batch correction. Seven types of cells were identified according to the annotation of the R package singleR along with the cell markers from the literature (22), including CCA cells, macrophages, CD8+ T cells, endothelial cells, fibroblasts, B cells and monocytes (**Figure 3F**). The estimation of the score of “Estrogen response late” for single cells *via* the AUCell method showed that the CCA cells exhibited the strongest estrogen response (**Figure 3G**, **Supplementary Figure 4A**). Then, we figured the expression of COMT and HSD17B1 in different cell types within CCA tissues to confirm the responsible cell types. It was shown that COMT was highly expressed in almost all cell types except for CD8^+^ T cells and HSD17B1 was mainly expressed in part of CCA cells, indicating CCA cells may directly regulate the estrogen response (**Supplementary Figures 4C, D**). Then, the CCA cells were further divided into 6 subgroups (**Figure 3H**). The estrogen response score also differed among the 6 subgroups (**Supplementary Figure 4B**). Transient cell states often followed with dynamic regulation of gene expression. Single cell RNA-seq helps in placing the cells on a hypothetical time trajectory that reflects gradual transition of their transcriptomes and also called pseudotime (32). The cell trajectory of CCA cells was plotted and annotated by cell clusters and pseudotim (**Figures 3I, J**). Interestingly, the estrogen response score significantly correlated with pseudotime (**Figure 3K**, Spearman correlation test r = 0.72, p < 2.2×10^-16^), indicating the potential links between the estrogen response and differentiation of CCA cells. KRT19 is considered to be a cholangiocyte/stem-cell marker (33, 34). Annexin A4 (ANXA4) is a marker of epithelial cell polarity (35). It seems that the expression of KRT19 was positively correlated and the expression of ANXA4 was negatively correlated with the estrogen response (**Supplementary Figure 4E–H**). Moreover, KRT19 increased and ANXA4 decreased following pseudotime (**Supplementary Figures 4I, J**). In summary, we hypothesized that the estrogen response is higher in cancer stem cells (CSCs) and may also be involved in cancer stemness acquisition. In addition, we found that the expression of COMT and HSD17B was also increased following pseudotime (**Figure 3L**).

### The Immune Microenvironment Differs Between Different ES Clusters

**Figure 4:**
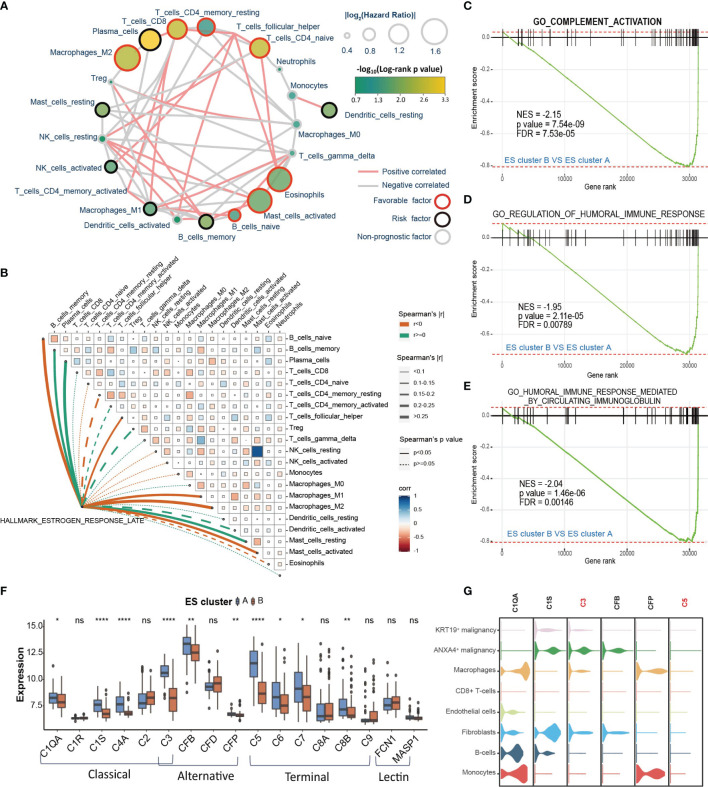
The immune microenvironment differs between different ES clusters. **(A)** Landscape of immune cells in CCA. The size of the circle represents the |log2HazardRatio| of each immune cell. The color filling the circle represents the -log_10_(log-rank p value). The color of the border represents the prognostic value of the immune cells, and the color of the line between two immune cells represents the cell interaction estimated by Spearman correlation analysis. **(B)** Correlation analysis among the GSVA score of “Estrogen response late” and infiltration of different immune cells. **(C–E)** GSEA plot showing that complement activation and humoral immune response are downregulated in ES cluster B versus ES cluster A **(F)** The expression of complement of different complement activation pathways in ES clusters A and B. **(G)** The expression of specific complements in different cell types of CCA tissues. Statistical p-value (*,P < 0.05; **,P < 0.01; ****,P < 0.0001). ns, not significant.

The functional analysis of ESRGs and GSEA results between ES clusters indicated that the immune microenvironment might be different between ES cluster A and B. Therefore, first, we established the landscape of immune cells in CCA. The immune filtration of 22 immune cells in 118 CCA samples of the GSE89749 cohort was estimated utilizing the cibersort algorithm. The hazard ratio of each cell type and the correlation between different cell types were calculated (**Figure 4A**). M2 macrophages, CD8^+^ T cells, CD4^+^ memory T cells, CD4^+^ naïve T cells, eosinophils, activated mast cells, and naïve B cells were favorable prognostic factors, and plasma cells, resting mast cells, activated NK cells, CD4^+^ activated memory T cells, M1 macrophages, activated dendritic cells, memory B cells and resting dendritic cells were risk prognostic factors. Then, we compared the abundance of these cells between ES cluster A and B. M2 macrophages and M1 macrophages decreased in ES cluster B, and CD4^+^ activated memory T cells, plasma cells and activated dendritic cells increased in ES cluster B (**Supplementary Figure 5A**). However, the immune score evaluated by the estimation algorithm between these two clusters showed no significant difference. (Data not shown) Among these differential immune cells, M2 macrophages were the top favorable factors, and plasma cells were the top risk factors, with the most significant difference between ES cluster A and B. Spearman correlation analysis also showed that the estrogen response was positively correlated with the abundance of plasma cells and negatively correlated with the abundance of M2 macrophages or M1 macrophages (**Figure 4B**). Interestingly, both plasma cells and macrophages are major components of humoral immunity, and aberrant humoral immunity is associated with tumor development (36). Several biological processes concerning humoral immunity were significantly downregulated in ES cluster B, such as “complement activation”, “regulation of humoral immune response” and “humoral immune response mediated by circulating immunoglobulin” (**Figures 4C** **–E**). It is well known that complement activation is an important part of antibody opsonization, and in terms of that the core enrichment of humoral immunity processes were mainly complement components, we supposed that the complement activation is the main difference between ES cluster A and B.

The role of the complement system in tumor progression is sophisticated. It may directly eliminate antibody-coated tumor cells or mediate an immunosuppressive environment supporting tumor development. The activation of complement consists of three pathways, including the classical pathway, lectin pathway and alternative pathway, which could further initiate the terminal pathway and the formation of the membrane attack complex (MAC) (37). Then, we analyzed the differential expression of the key genes in these four pathways between ES cluster A and B. The expression of C1QA, C1S, C4A, C3, CFB, CFP, C5, C6, C7 and C8B was downregulated, and no gene was upregulated in ES cluster B (**Figure 4F**). The genes in the classical pathway shared the greatest difference, and C3 and C5 were the top differentially expressed genes between the two groups. Then, we attempted to examine complement activation at the single-cell level. Considering the distinction between KRT19^+^ and ANXA4^+^ CCA cells were then subdivided into KRT19^+^ and ANXA4^+^ subtypes (**Supplementary Figure 5B**). We scored the complement activation process with the AUCell method and found that complement activation was most significant in macrophages and monocytes (**Supplementary Figures 5C, D**). Therefore, we supposed that the decrease in macrophages may partially account for the downregulation of complement activation in ES cluster B. In addition, it was surprising that the level of complement activation in ANXA4^+^ CCA cells ranked second only to macrophages and monocytes. Then, we checked the expression of these differentially expressed genes at the single-cell level (**Figure 4G**). The expression of C3 and C5 was highest in ANXA4^+^ CCA cells and hardly expressed in KRT19^+^ CCA cells. Moreover, C1S was mainly expressed in ANXA4^+^ CCA cells, and C1QA and CFP were mainly expressed in macrophages and monocytes. Meanwhile, we investigated the difference in activity of complement activation between CCA tumor and non-tumor tissues. Surprisingly, “GO_COMPLEMENT_ACTIVATION” was significantly downregulated in CCA tissues, and all the key genes in the four complement activation pathways were significantly declined (**Supplementary Figures 5E, F**). Finally, the results showed that both the expression of C3 and C5 could predict good prognosis (**Supplementary Figures 5G, H**, C3: HR = 0.442, log-rank p value = 0.0036; C5: HR = 0.338, log-rank p value = 0.00011).

### Establishment and Validation of the Estrogen Response-Related Score in CCA Patients

**Figure 5:**
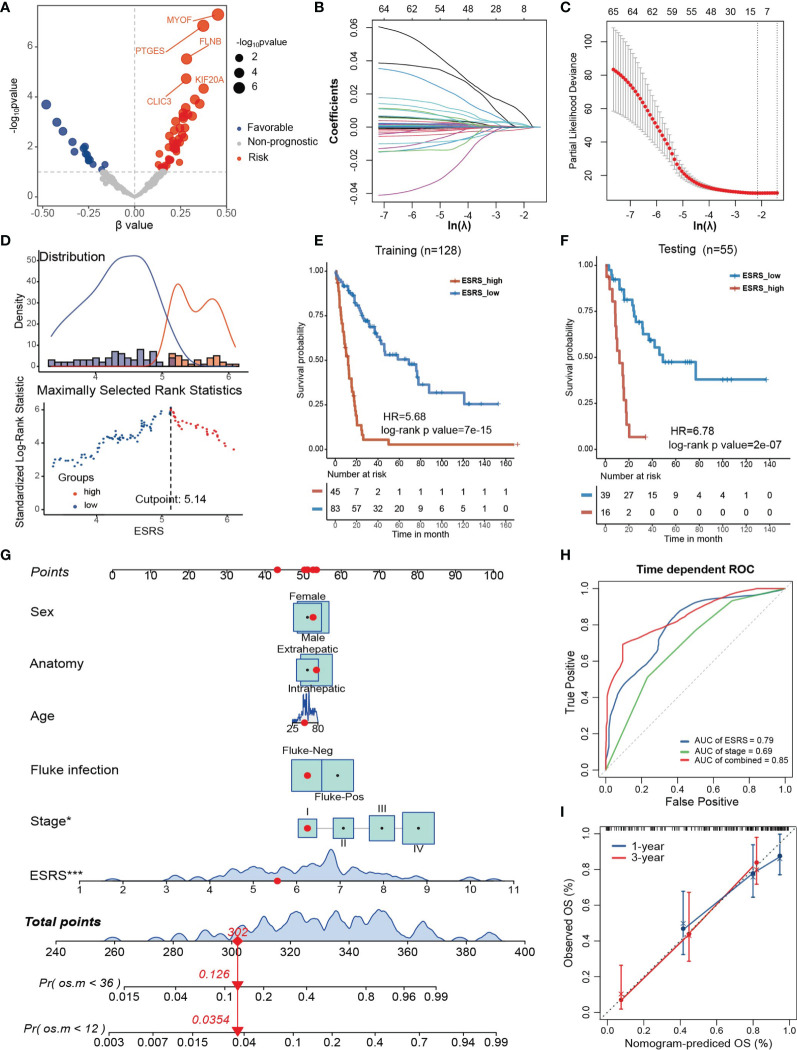
Establishment and validation of the estrogen response-related score (ESRS) in CCA patients. **(A)** Volcano plot showing the univariate Cox regression results of the estrogen response-related genes in the meta-cohort. The horizontal coordinate represents the coefficient of univariate Cox regression, and the vertical coordinate represents the -log10p value of univariate Cox regression. **(B)** With a filtering threshold of a P value less than 0.1, 65 candidates remained and were entered into the LASSO Cox regression model. **(C)** Tenfold cross-validation for tuning parameter selection in the LASSO model. The dotted vertical lines are drawn at the optimal values by minimum criteria (lambda.min, left vertical dotted line) and 1-SE criteria (lambda.1 se, right vertical dotted line). **(D)** The best cutoff value of ESRS was chosen by maximally selected rank statistics. **(E)** Kaplan–Meier analysis demonstrated that patients with higher ESRS exhibited worse prognosis in the training cohort (HR = 5.68, log-rank p value = 7×10^-15^). **(F)** In the testing cohort, Kaplan–Meier analysis showed that patients with higher IRS exhibited worse prognosis (HR = 6.78, log-rank p value = 2×10^-7^). **(G)** A personalized scoring nomogram was generated to predict 3- and 5-year OS probability with the seven parameters, and the red arrow shows an example. **(H)** Time-dependent ROC analysis demonstrated that combining ESRS and stage information could better predict the 5-year OS probability. **(I)** Calibration curves of 1-year and 3-year OS prediction were close to the ideal performance (45-degree line). Statistical p-value (*,P < 0.05; ***,P < 0.001).

Because the estrogen response varies among different clinicopathological features and stably correlates with poor prognosis, we attempted to construct a prognostic model based on genes in “Estrogen response late” to predict overall survival probability in CCA. First, the GSE89749 cohort and E-MTAB-6389 cohort were merged into meta-data, followed by batch correction. The TCGA-CHOL cohort was dismissed due to incompatible RNA sequencing data and a small sample size. The merged data set was further randomly divided into a training cohort (n = 128) and a testing cohort (n=55). Then, univariate Cox regression analysis was performed for each gene in “Estrogen response late” (**Figure 5A**). Sixty-five genes with univariate Cox regression p values less than 0.1 were subsequently entered into the LASSO Cox regression model (**Figure 5B**). Tenfold cross-validation was then employed to tune parameter selection in the LASSO model, and 11 genes, including ANXA9, NRIP1, FKBP5, PDLIM3, GJB3, MYOF, PTGES, ID2, CDC20, MDK and SNX10, were filtered to construct the ESRS *via* multivariate Cox regression analysis (**Figure 5C**). The best cutoff value, used to divide the training cohort into ESRS_high and ESRS_low groups, was chosen by maximally selected rank statistics (**Figure 5D**). It was shown that the ESRS did an excellent job in predicting prognosis in the training cohort (**Figure 5E**, HR = 5.68, log-rank p value= 7×10^-15^). To test the stability of the ESRS model, the computational formula along with the best cutoff value was applied in the testing cohort. The ESRS also performed well in predicting overall survival probability in the testing cohort (**Figure 5F**, HR = 6.78, log-rank p value= 2×10^-7^)

To improve the risk stratification and personalized assessment of the prognostic model, we integrated the ESRS with the clinical information of CCA patients. In total, 98 samples with full clinical information on age, sex, fluke infection status, anatomy and clinical stage in the GSE89749 cohort were selected for the construction of the integrated prognostic model. Next, a nomogram was established based on the selected clinical information and ESRS, which could provide personalized and convenient assessment of 3-year and 5-year survival probability. The red arrow is an example (**Figure 5G**). As shown in the nomogram, ESRS and the clinical stage of CCA patients were independent and stable prognostic factors in CCA patients. Time-dependent ROC curves indicated that combining clinical stage could improve the predictive ability of ESRS for OS (**Figure 5H**). Finally, to check the validity of the nomogram, we drew calibration curves for the prediction of 1-year and 3-year OS, which were closely correlated with the ideal performance, indicating the accuracy of the prediction of the nomogram (**Figure 5I**). Altogether, the integrated prognostic model could accurately and effectively predict the OS of CCA patients.

### Potential Therapeutic Targets and Applicable Drugs According to ESRS or Estrogen Response

**Figure 6:**
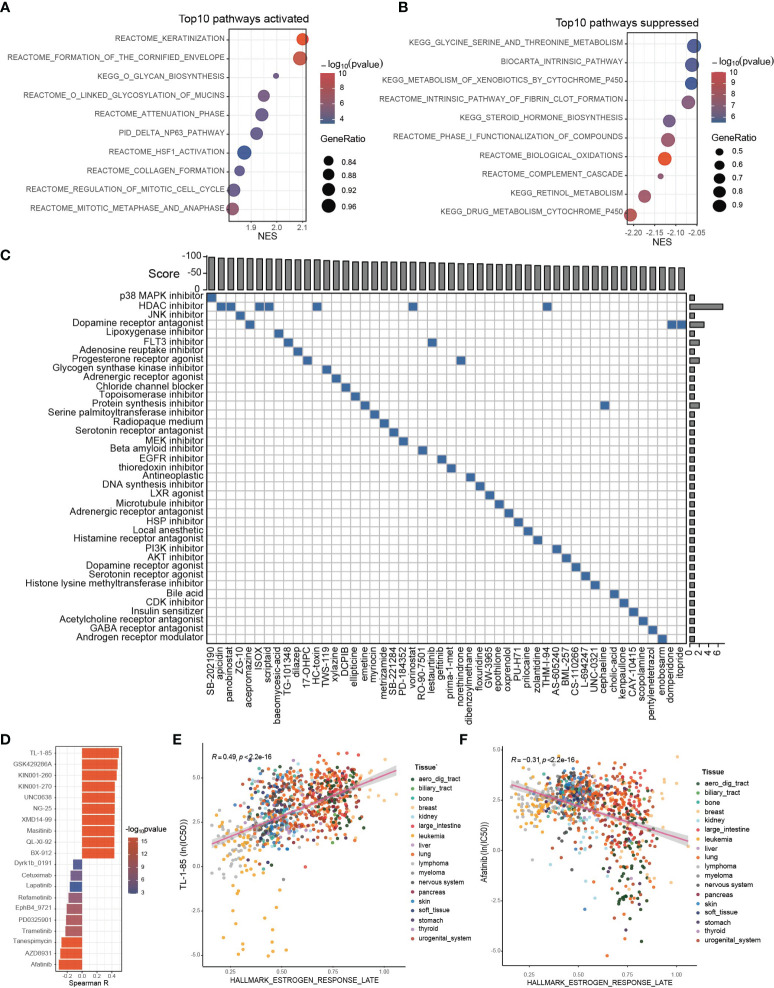
Potential therapeutic targets and applicable drugs according to ESRS or estrogen response. **(A, B)** GSEA was performed between the ESRS_high and ESRS_low groups. The bar plots show the top 10 upregulated or downregulated pathways in the ESRS_high group. **(C)** The differentially expressed genes were submitted to CMap mode-of-action (MoA) analysis, and the results showed 39 mechanisms of action shared by the top 50 compounds potentially applicable for ESRS_high patients. **(D)** The correlation between GSVA score of “Estrogen response late” and ln(IC50) of drugs in cancer cell lines. **(E, F)** Scatter plots showing the correlation between the GSVA score of “Estrogen response late” and the ln(IC50) of TL-1-85 or afatinib in cancer cell lines. The color of the dot represents the histologic origin of the cancer cell lines.

Since the estrogen response and the established ESRS are closely correlated with CCA progression, we attempted to explore personalized chemotherapeutic agents or molecular targeted therapeutic drugs for individuals, taking the estrogen response as a clue. First, we performed GSEA for the differentially expressed genes between the ESRS_high and ESRS_low groups to identify the potential pathways involved in CCA progression. The top enriched pathway in the ESRS_high group was “keratinization” (**Figure 6A**; **Supplementary Figure 6A**). A protein–protein interaction network (PPI) was constructed based on the differentially expressed genes in the gene set “Keratinization” utilizing the STRING database (**Supplementary Figure 6B**). Desmoglein 3 (DSG3) was identified as the core gene of the network to the highest degree. Similarly, “drug metabolism cytochrome P450” was the top enriched pathway in the ESRS_low group (**Figure 6B**; **Supplementary Figure 6C**). Cytochrome P450 Family 2 Subfamily C Member 9 (CYP2C9) and Cytochrome P450 Family 3 Subfamily A Member 7 (CYP3A7) were identified as core genes in the PPI network of “Drug metabolism cytochrome P450” (**Supplementary Figure 6D**). These identified pathways and core genes could be potential targets for drug development.

Thanks to previous efforts, CMap, a map of disease-gene-drug connections, has been constructed, which takes differential gene expression as a common language of disease and chemical compounds (38, 39). We submitted a list of 150 upregulated and 150 downregulated genes between the ESRS_high and ESRS_low groups to the mode-of-action (MoA) analysis of CMap to search for chemical compounds that could lead to similar or reverse genomic fluctuations (**Supplementary Table S3**). 39 mechanisms of action were shared by the top 50 compounds potentially applicable for ESRS_high patients, which could cause reverse changes between the ESRS_high and ESRS_low groups (**Figure 6C**). In particular, SB-202190, a p38 MAPK inhibitor, ranked first among all the potential compounds, and 7 potential compounds, namely, apicidin, panobinostat, ISOX, scriptaid, HC-toxin, vorinostat and THM-I-94, were all histone deacetylase (HDAC) inhibitors. In addition, we also showed 42 mechanisms of action shared by the top 50 compounds that show similar impacts on cancer cells to ESRS, which should be dismissed for ESRS_high patients (**Supplementary Figure 6E**).

Drug resistance has always been the principal factor impeding satisfactory cures for patients with cancer, for which tumor heterogeneity may be partially blamed (40, 41). To optimize drug selection, we correlated the IC50 of chemical drugs with the GSVA score of “Estrogen response late” in all cancer cell lines from GDSC (**Figure 6D**; **Supplementary Table S4**). Patients with a high estrogen response may be more likely to acquire resistance to drugs with a positive correlation with the estrogen response and more sensitive to drugs with a negative correlation with the estrogen response. Among them, the IC50 of TL-1-85 was the top drug positively correlated with the estrogen response (**Figure 6E**, Spearman correlation test, r = 0.49, p < 2.2×10^-16^). The IC50 of afatinib was the top drug negatively correlated with the estrogen response (**Figure 6F**, Spearman correlation test, r = -0.31, p < 2.2×10^-16^).

## Discussion

Estrogen has been proven to be involved in cancer progression by modulating proliferation, apoptosis, epithelial-to-mesenchymal transition (EMT), angiogenesis, tumor metabolism and the immunosuppressive microenvironment (42–47). Therapeutics targeting selective estrogen receptor or estrogen signaling have been developed and have achieved satisfactory clinical outcomes, especially in breast cancer (48, 49). While it has long been established that CCA could be regulated by estrogen, current knowledge about the mechanism of estrogen signaling regulating CCA progression is still in its infancy. Let alone exploration for potential therapeutic targets specific to the heterogeneity of estrogen response.

We were the first to characterize the clinical and transcriptomic landscape of the estrogen response in CCA. In our study, it was shown that the estrogen response differs among different cancer types and that the estrogen response predicted the worst prognosis in patients with CCA. WGCNA was conducted to identify the ESRGs, based on which patients with CCA were clustered into ES cluster A and B. The clinical features, biological characteristics and estrogen metabolism were significantly different between the two clusters. Single-cell level analysis indicated that the estrogen response was strongest in CCA cells and possibly correlated with cancer stemness acquisition. The immune landscape in CCA was constructed, and it was shown that the estrogen response was positively correlated with the infiltration of plasma cells and negatively correlated with that of macrophages. The activation of complement was significantly downregulated in ES cluster B, and the decrease in C3, C5 and C1S in CCA cells may be a potential cause. We also established an ESRS prognostic model based on the expression of estrogen response-related genes and integrated the ESRS with clinical features to construct a nomogram. Finally, we constructed a pharmacological landscape specific to the estrogen response in patients with CCA, taking advantage of CMap and the GDSC database.

In the GSE89749 cohort, ES cluster B with an intense estrogen response was mainly composed of patients with fluke infection. Estrogen is well known for its anti-inflammatory effect (26, 50). Meanwhile, the main risk factors to date are fluke infection and primary sclerosing cholangitis, both of which could give rise to a chronic inflammatory environment (2). In addition, the liver is the primary site for estrogen metabolism (30, 51), and substantial evidence has indicated that chronic liver inflammation is accompanied by elevated estrogen levels and endocrine disturbance, potentially on account of the impaired function of liver cells to inactivate estrogen (25, 52, 53). Therefore, we hypothesized that chronic inflammation and elevated estrogen response accompany each other and are involved in the tumorigenesis and progression of CCA. The ES clusters also overlapped with the classification of the International Cancer Genome Consortium (10). ES cluster A was mainly composed of CCA cluster 3/4 characterized by high copy number alterations and expression of PD-1/PD-L2 or epigenetic changes (IDH1/2, BAP1) along with FGFR/PRKA-related gene rearrangements. ES cluster B was composed of CCA cluster 1/2, characterized by ERBB2 amplifications and TP53 mutations. Therefore, ES clusters may provide a reference in the development and selection of specific chemotherapeutic targets in CCA, such as therapies targeting ERBB2/HER2 signaling (54), IDH inhibitors (ClinicalTrials.gov identifier: NCT02073994) and RGFR-targeting agents (55).

The differential estrogen response could be derived either from the different expression of estrogen receptors or the disturbance in estrogen metabolism. For example, genetic variations in COMT may influence the risk of breast cancer as a result of significant changes in catechol estrogen and methoxyestrogen levels (56). Studies have shown that five cytochrome P450 1B1 (CYP1B1) variants, which could change the hydroxylation activity of estrogen, have greater than twofold higher activity than the wild-type enzyme, thereby increasing the risk for cancers (57). The expression of HSD17B2, which catalyzes estradiol (E2) to E1 in human CRC tissue, was also proven to be downregulated in colon cancers and predicts poor prognosis, suggesting an important role of estrogen metabolism in CRC progression (58). In our study, we found that COMT, which is responsible for the inactivation of estrogen, was negatively correlated with the estrogen response and HSD17B1, which catalyzes E1 to more active E2, was positively correlated with the estrogen response. Meanwhile, COMT correlates with good prognosis, and HSD17B1 predicts poor prognosis. These findings strongly suggest the important role of these two genes in estrogen metabolism in CCA and provide potential targets for CCA treatment. In addition, we also found that the estrogen response was correlated with cancer cell differentiation and cancer stemness, marked by KRT19, in CCA, which deserves further investigation.

Complement plays a key role in the innate immune system and defense against pathogens and is also a neglected component of the TME derived from tumor cells, infiltrated cells or the circulation (37). The role of the complement system in cancer progression is crucial and complex. For example, C1q can also exert antitumoural effects by induction of apoptosis in breast or prostate cancer cell lines (59, 60). While C5a exerts immunosuppression effect by recruiting MDSCs to the TME, which in turn suppress effector T cells (61). In our study, we found that downregulation of complement activation was a representative feature of ES cluster B. Further investigation indicated that most of the complement components were decreased in ES cluster B, including C1QA, C1S, C4A, C3, CFB, CFP, C5, C6, C7 and C8B. Single-cell level analysis revealed that the expression of C3 and C5 was highest in ANXA4^+^ CCA cells and hardly expressed in KRT19^+^ CCA cells, both of which were correlated with good prognosis in patients with CCA. Interestingly, the downregulation of complement activation in CCA tumor tissues compared with CCA non-tumor tissues was even more significant. Our findings suggested that complement activation may play a protective role in patients with CCA, and the downregulation of complement activation may be attributed to an upregulated estrogen response, which needs further investigation. Many agents targeting complement are in the pipeline for various diseases, including cancer therapy (62–64). For example, IgG hexamerization was designed to promote C1q binding and complement activation and enhance the anticancer effect (65). Our findings of complement in CCA may provide insights into the exploration of relevant chemotherapeutic agents.

Several prognostic models have been established in CCA based on signatures of mRNA, DNA methylation or alternative splicing (66–68). These prognostic models could help predict overall or disease-free survival and are beneficial to the individual management of CCA patients. In our study, we constructed an ESRS model based on the expression of estrogen response-related genes, which performed well in predicting the prognosis of patients with CCA and performed well in both the training and testing cohorts. PPI networks concerning “keratinization” and “drug metabolism cytochrome P450” were constructed to find potential gene targets for the ESRS_high group. Then, the CMap database was employed to identify potential chemotherapeutic agents for the ESRS_high group. Among them, SB-202190, a p38 MAPK inhibitor, ranked at the top of recommended drugs, and p38 MAPK signaling has been reported to be a downstream signal of estrogen (69). In addition, 7 HDAC inhibitors were on the recommended list. It was reported that the addition of the HDAC inhibitor vorinostat (VPA), which is also in the recommended list, to ER-positive breast cancer cells could significantly increase the efficacy of tamoxifen treatment (70). Thus, we supposed that the drug list for the ESRS_high group was trustworthy. Furthermore, we also scanned all the chemotherapy drugs to make a list of drugs sensitive or resistant to CCA with a high estrogen response employing the data from the GDSC database.

Despite comprehensive characterization of the estrogen response, the preliminary research basis is insufficient. We obtained thrilling results, while the explanation for the findings was mainly based on speculation. Further clinical trials and experiments are needed to illuminate our findings. In addition, the size of the samples with available clinical information was limited, and the source of the datasets was diverse, which may cause bias of the results. Finally, further multiomics analyses, such as copy number variation, DNA methylation, and gene mutation, may promote an in-depth understanding of the role of estrogen in CCA progression and broaden the range of potential molecular targets.

In summary, our study characterized the landscape of the estrogen response in the clinical features, transcriptome and TIME of CCA. Estrogen response-based classification and risk models were established to predict prognosis and optimize individual management for patients with CCA. In addition, our results also provide applicable drugs and potential therapeutic targets in estrogen metabolism, the complement system and ESRS-related pathways for CCA.

## Data Availability Statement

The authors declare that the original contributions presented in the study are included in the article/**Supplementary Materials**, and further inquiries can be directed to the corresponding authors.

## Author Contributions

Conception and design of the study: SB, JC and HZ. Data collection and preprocessing: ML, QZ. Analysis and interpretation of data: CL, JM and QZ. Manuscript writing: CL, JM and ML. Visualization, statistical expertise: FX, YP, YW, ZY, XX. All authors contributed to the article and approved the submitted version.

## Funding

The Nanjing Health Science and Technology Development Special Fund for Distinguished Young Scholar [No. JQX19001], the National Science Foundation for Young Scholars of China [No. 81501380] and the Suzhou Science and Education Guardian Youth Science and Technology Project [No. KJXW2017065].

## Conflict of Interest

The authors declare that the research was conducted in the absence of any commercial or financial relationships that could be construed as a potential conflict of interest.

## Publisher’s Note

All claims expressed in this article are solely those of the authors and do not necessarily represent those of their affiliated organizations, or those of the publisher, the editors and the reviewers. Any product that may be evaluated in this article, or claim that may be made by its manufacturer, is not guaranteed or endorsed by the publisher.
