# Supplementary material for: Characterization of the Estrogen Response Helps to Predict Prognosis and Identify Potential Therapeutic Targets in Cholangiocarcinoma
Source: Front Oncol. 2022 May 19;12:870840. doi: 10.3389/fonc.2022.870840 (PMC9162778; doi:10.3389/fonc.2022.870840)
Supplement: Supplementary file 1 [file DataSheet_1.pdf]

Supplementary Table-S1: Basic information of datasets engaged in this study

Source/Accession number	Platform	Number of samples	Stage	Sex	age	Survival data	Organization
GEO: GSE26566	Illumina humanRef-8 v2.0 expression beadchip	Surrounding liver: n = 59<br>Normal intrahepatic bile duct: n = 6<br>Cholangiocarcinoma: n = 104	—	—	—	—	Biotech Research and Innovation Centre, Copenhagen University
GEO: GSE33327	Illumina HumanRef-8 WG-DASL v3.0	normal biliary epithelial cells: n = 6<br>intrahepatic cholangiocarcinoma: n = 149	—	—	—	—	University of Texas Southwestern Medical Center
GEO: GSE76297	Affymetrix Human Transcriptome Array 2.0	HCC Non-Tumor Tissue: n = 59<br>HCC Tumor Tissue: n = 62<br>CCA Non-Tumor Tissue: n = 92<br>CCA Tumor Tissue: n = 91	—	—	—	—	Liver Carcinogenesis Unit, Laboratory of Human Carcinogenesis, National Cancer Institute
GEO: GSE89749	Illumina HumanHT-12 V4.0 expression beadchip	CCA Non-Tumor Tissue: n = 2<br>CCA Tumor Tissue: n = 118	I: 14; II: 17; III: 26; IV: 41; NA: 20	M: 65, F:53	58,[26,79]	os	CMR, National Cancer Centre Singapore
ArrayExpress：E-MTAB-6389	Affymetrix GeneChip HTA-2_0	Cholangiocarcinoma: n = 78	—	M: 38, F:40	—	os	the french Ligue Nationale Contre le Cancer
TCGA-CHOL	Illumina RNAseq	CCA Non-Tumor Tissue: n = 9<br>Cholangiocarcinoma: n = 36	I: 19; II: 12; III: 5	M: 20, F:16	63,[29,89]	os,rfs	National Cancer Institute
GEO:GSE138709	HiSeq X Ten	31,302 cells of five ICC samples, as well as three paired adjacent non-tumor tissues	—	—	—	—	The Fifth Medical Center of Chinese PLA General Hospital, Academy of Military Medical Sciences

Supplementary Table-S2: Clinical infromation, ES clusters, ES-related score of CCA patients in the GSE89749 cohort

id	os	os.m	Fluke-infection	Country	Sex	Age at surgery	Anatomical subtype	Primary Sclerosing cholangitis	HBV	HCV	Histology	TNM staging	Stage	CCA cluster	GSVA score of Estrogen response	ES cluster	ESRS
CCA_BR_1	1	0.866666667	Fluke-Neg	Brazil	M	53	Intrahepatic	No	Negative	Negative	Moderate	T3NxMx	NA	4	0.464562968	A	2.084593182
CCA_BR_2	1	18.86666667	Fluke-Neg	Brazil	F	49	Intrahepatic	No	Negative	Negative	Moderate	T3N0M1	IVB	4	0.49568426	A	5.526928303
CCA_FR_1	0	68.13333333	Fluke-Neg	France	F	49	Intrahepatic	No	Negative	Negative	Poor	T1NxM0	NA	4	0.484735338	A	4.411290261
CCA_FR_2	0	10.93333333	Fluke-Neg	France	M	78	Intrahepatic	No	Negative	Negative	Moderate	T3NxM0	NA	4	0.505254325	A	4.345722658
CCA_FR_3	0	9.333333333	Fluke-Neg	France	F	60	Intrahepatic	No	Negative	Negative	Poor	T3NxM0	NA	1	0.503807471	B	6.434564218
CCA_FR_4	1	5.266666667	Fluke-Neg	France	M	75	Intrahepatic	No	Negative	Negative	Poor	T3N1M0	IVA	4	0.458601186	A	3.555729196
CCA_FR_7	0	8.266666667	Fluke-Neg	France	F	77	Intrahepatic	No	Negative	Negative	Well	T3N1M0	IVA	4	0.51566985	A	4.537377916
CCA_FR_8	0	74.16666667	Fluke-Neg	France	F	73	Intrahepatic	No	Negative	Negative	Moderate	T3NxM0	NA	4	0.497870229	A	3.54755393
CCA_FR_9	0	56.3	Fluke-Neg	France	M	62	Intrahepatic	No	Negative	Negative	Moderate	T4NxM0	IVA	4	0.456483058	A	5.16469086
CCA_FR_10	0	107.2333333	Fluke-Neg	France	M	29	Intrahepatic	Yes	Negative	Negative	Well	T3N1M0	IVA	3	0.476350827	A	4.297375293
CCA_FR_11	0	103.8	Fluke-Neg	France	M	45	Intrahepatic	No	Negative	Negative	Well	T2N0M0	II	4	0.449543909	A	4.511203413
CCA_FR_12	0	8.9	Fluke-Neg	France	F	43	Intrahepatic	No	Negative	Negative	Moderate	T4NxM0	IVA	4	0.488822786	A	4.882744991
CCA_FR_13	1	46.7	Fluke-Neg	France	M	51	Intrahepatic	No	Negative	Negative	Moderate	T3NxM0	NA	4	0.499973632	A	4.953720745
CCA_FR_14	0	7.3	Fluke-Neg	France	F	52	Intrahepatic	No	Negative	Positive	Well	T3NxM0	NA	2	0.51763913	A	4.754637961
CCA_FR_15	0	51.03333333	Fluke-Neg	France	M	56	Intrahepatic	No	Negative	Negative	Well	T4N0M0	IVA	4	0.472143628	A	2.904744888
CCA_FR_16	0	0.633333333	Fluke-Neg	France	F	78	Intrahepatic	No	Negative	Negative	Moderate	T3NxM0	NA	3	0.456527927	A	4.943644691
CCA_KR_2	1	25.9	Fluke-Neg	Korea	M	70	Intrahepatic	No	Negative	Negative	Moderate	T2bN0	NA	4	0.465281557	A	4.662628148
CCA_RO_2	1	20.1	Fluke-Neg	Romania	F	49	Perihilar	No	Negative	Negative	NA	T2N0M1	IV	1	0.497832041	B	5.715438506
CCA_RO_3	1	3.1	Fluke-Neg	Romania	M	46	Perihilar	No	Negative	Negative	Moderate-Po	T3N1M0	IIB	2	0.468291399	B	5.623893832
CCA_RO_4	1	NA	Fluke-Neg	Romania	F	53	Intrahepatic	No	NA	NA	NA	NA	NA	2	0.526221098	B	6.047015367
CCA_RO_5	1	0.3	Fluke-Neg	Romania	F	69	Intrahepatic	No	Negative	Negative	Well	NA	NA	4	0.469211603	A	4.666716092
CCA_RO_6	1	NA	Fluke-Neg	Romania	M	NA	Intrahepatic	No	NA	NA	NA	NA	NA	4	0.47379786	A	4.023974872
CCA_RO_7	1	76.8	Fluke-Neg	Romania	M	68	Distal	No	Negative	Negative	Well	T1N1M0	IA	2	0.547744729	B	5.459795859
CCA_RO_10	0	28.26666667	Fluke-Neg	Romania	F	61	Intrahepatic	No	Negative	Negative	poor	T3N0M0	III	4	0.453546703	A	4.198006835
CCA_RO_15	0	57.4	Fluke-Neg	Romania	F	61	Perihilar	No	Negative	Negative	Well-Modera	T3N0M0	IIIA	4	0.467462715	A	4.150248462
CCA_RO_19	1	17.96666667	Fluke-Neg	Romania	F	66	Intrahepatic	No	Negative	Negative	Moderate	T1NXM0	NA	2	0.517364348	A	5.146909448
CCA_RO_23	1	10.03333333	Fluke-Neg	Romania	F	53	Perihilar	No	Negative	Negative	Well	T4N1M0	III	2	0.558388292	B	6.073481388
CCA_RO_25	1	12.06666667	Fluke-Neg	Romania	M	78	Intrahepatic	No	Negative	Positive	Well	T3NXM0	NA	3	0.524464928	A	5.420379105
CCA_RO_29	1	40.33333333	Fluke-Neg	Romania	M	72	Perihilar	No	Negative	Negative	Well	T2N0M0	II	2	0.530818016	B	4.72514818
CCA_RO_31	1	24.5	Fluke-Neg	Romania	M	58	Distal	No	Negative	Negative	Well	T1N1M0	IIB	2	0.534791107	B	5.307821262
CCA_RO_35	1	15.73333333	Fluke-Neg	Romania	M	62	Intrahepatic	No	Negative	Negative	Moderate	T2bNxM0	NA	2	0.525425373	A	6.248080872
CCA_RO_38	1	19.16666667	Fluke-Neg	Romania	F	44	Intrahepatic	No	Negative	Negative	Moderate	T2bN1M0	IVA	4	0.477162565	A	4.885494704
CCA_RO_42	1	50	Fluke-Neg	Romania	M	70	Distal	No	Negative	Negative	Moderate	T3N0M0	IIA	2	0.552650712	B	6.0159382
CCA_RO_43	1	7	Fluke-Neg	Romania	M	40	Intrahepatic	Yes	NA	NA	Well	T3N2M0	IVA	NA	0.565937999	B	5.01140575
CCA_RO_45	1	9.2	Fluke-Neg	Romania	M	69	Intrahepatic	No	NA	NA	Well	NA	NA	NA	0.479129639	A	4.879854746
CCA_RO_46	1	13.96666667	Fluke-Neg	Romania	M	69	Distal	No	Negative	Negative	Well	T3N1M0	IIB	2	0.54371031	B	5.04706508
CCA_SG_1	0	7.733333333	Fluke-Neg	Singapore	F	73	Intrahepatic	No	Positive	Negative	Moderate	T3N0M0	III	2	0.489387071	A	4.842582723
CCA_SG_2	0	38.46666667	Fluke-Neg	Singapore	F	67	Intrahepatic	No	Positive	Negative	Moderate	T1N1M0	IVA	4	0.453264399	A	4.062759908
CCA_SG_3	0	28.6	Fluke-Neg	Singapore	F	51	Intrahepatic	No	Negative	Negative	Moderate	T1N0M0	I	4	0.458986315	A	3.717525738
CCA_SG_4	0	94.93333333	Fluke-Neg	Singapore	M	61	Intrahepatic	No	Positive	Negative	Well	T1N0M0	I	4	0.451397618	A	3.673028887
CCA_SG_5	0	17.43333333	Fluke-Neg	Singapore	F	78	Intrahepatic	No	Negative	Negative	Moderate	T1N1M0	IVA	2	0.508578009	B	6.087022314
CCA_SG_6	1	46.03333333	Fluke-Neg	Singapore	M	51	Intrahepatic	No	Negative	Negative	Moderate	T1N0M0	I	4	0.510778366	A	5.408535316
CCA_SG_7	0	29.93333333	Fluke-Neg	Singapore	M	68	Extrahepatic	No	Negative	Negative	Moderate-Po	T1N0M0	I	2	0.566069578	B	5.608943731
CCA_SG_8	0	71.66666667	Fluke-Neg	Singapore	M	57	Intrahepatic	No	Positive	Negative	Poor	T1N0M0	I	3	0.497980351	A	4.04125413
CCA_SG_9	1	3.033333333	Fluke-Neg	Singapore	F	60	Intrahepatic	No	Negative	Negative	Moderate	T1N0M1	IVB	4	0.424125335	A	4.161421558
CCA_SG_10	0	28.03333333	Fluke-Neg	Singapore	M	74	Intrahepatic	No	Negative	Negative	Moderate	T1N0M0	I	4	0.439702741	A	4.686951812
CCA_SG_11	0	17.23333333	Fluke-Neg	Singapore	F	69	Intrahepatic	No	Negative	Negative	Moderate	T2aN1M0	IVA	4	0.471290255	A	4.816751989

CCA_SG_12	0	51.33333333	Fluke-Neg	Singapore	F	61	Intrahepatic	No	Positive	Negative	Moderate	T1N0M0	I	4	0.447780403	A	4.242119951
CCA_SG_13	0	32.73333333	Fluke-Neg	Singapore	M	60	Intrahepatic	No	NA	NA	Well	T1N0M0	I	2	0.527045102	B	5.337116459
CCA_SG_14	0	37.13333333	Fluke-Neg	Singapore	F	61	Intrahepatic	No	Negative	Negative	Well	NA	NA	2	0.508794738	B	4.898216718
CCA_SG_15	0	33	Fluke-Neg	Singapore	M	53	Intrahepatic	No	No recor	No recor	Well	T3N0M0	III	4	0.47202849	A	3.791947128
CCA_SG_16	0	28.9	Fluke-Neg	Singapore	M	55	Intrahepatic	No	Positive	Negative	Moderate	T3N0M0	III	4	0.461254435	A	5.335924209
CCA_SG_17	1	18	Fluke-Neg	Singapore	F	77	Intrahepatic	No	NA	NA	NA	T2bN1M0	IVA	3	0.45979648	A	4.34293145
CCA_SG_18	0	4.3	Fluke-Neg	Singapore	F	71	Extrahepatic	No	Negative	Negative	Well	T3N0M0	IIA	2	0.539658347	B	4.826836077
CCA_SG_19	0	18.46666667	Fluke-Neg	Singapore	M	75	Intrahepatic	No	Negative	Negative	Moderate	T1aN0M0	II	4	0.442179929	A	4.236022931
CCA_SG_20	0	12.7	Fluke-Neg	Singapore	M	56	Intrahepatic	No	Negative	Negative	Well	T1N0M0	I	4	0.413928002	A	2.891752902
CCA_SG_21	0	42.06666667	Fluke-Neg	Singapore	F	55	Intrahepatic	No	Positive	Positive	Moderate	T4N0M0	IVA	4	0.474613447	A	4.22016697
CCA_SG_24	1	17.6	Fluke-Neg	Singapore	F	45	Perihilar	No	Negative	Negative	Moderate	T4N1M0	IVA	2	0.52328877	B	6.732303608
CCA_SG_25	0	37.63333333	Fluke-Neg	Singapore	F	74	Intrahepatic	No	Negative	Negative	Poor	T1N0M0	I	4	0.463839685	A	4.75119171
CCA_SG_29	0	13.53333333	Fluke-Neg	Singapore	F	39	Intrahepatic	No	Negative	NA	Poor	T1N0M0	I	3	0.489096504	A	5.015368307
CCA_SG_34	0	20.8	Fluke-Neg	Singapore	M	35	Perihilar	No	NA	NA	TBA	T3N0M0	III	4	0.411902345	A	4.476307155
CCA_SG_48	0	2.466666667	Fluke-Neg	Singapore	F	77	Perihilar	No	NA	NA	NA	TxN1Mx	NA	2	0.564337948	B	6.666842145
CCA_SG_49	0	66.3	Fluke-Neg	Singapore	M	49	Perihilar	Yes	NA	Negative	Moderate	T2bN0M0	II	4	0.448677149	A	4.279701757
CCA_SG_50	1	11.36666667	Fluke-Neg	Singapore	M	32	Intrahepatic	No	Negative	Negative	Poor	T2bN1M1	IVB	4	0.487224081	A	5.106460352
CCA_SG_51	0	13.83333333	Fluke-Neg	Singapore	M	63	Intrahepatic	No	Negative	Negative	Moderate	T2aN0M0	II	NA	0.497767601	A	4.787613621
CCA_SG_52	0	11.6	Fluke-Neg	Singapore	F	26	Intrahepatic	No	Negative	Negative	Moderate	T2bN0M0	II	NA	0.498356031	A	5.749038276
CCA_SG_54	0	7.733333333	Fluke-Neg	Singapore	F	60	Intrahepatic	No	NA	NA	Moderate	T3N1M1	IVB	4	0.462899493	A	4.257683181
CCA_TH_1	1	5.566666667	Fluke-Pos	Thailand	F	49	Intrahepatic	No	Positive	Negative	Moderate	T3N0M0	III	1	0.540743073	B	6.733194196
CCA_TH_2	1	20.83333333	Fluke-Pos	Thailand	M	46	Perihilar	No	NA	NA	Well	T3N0M0	IIIA	2	0.512552645	B	5.651869611
CCA_TH_3	1	14.66666667	Fluke-Pos	Thailand	F	76	Perihilar	No	NA	NA	Well	T3N0M0	IIIA	2	0.502627553	B	6.385036678
CCA_TH_4	1	1	Fluke-Pos	Thailand	M	53	Perihilar	No	NA	NA	Well	T3N1M1	IVB	NA	0.547149183	B	7.657964536
CCA_TH_5	1	12	Fluke-Pos	Thailand	M	60	Perihilar	No	NA	NA	Papillary c	T4N0M0	IVA	1	0.456474688	B	7.012218236
CCA_TH_6	1	5.133333333	Fluke-Pos	Thailand	F	51	Intrahepatic	No	Negative	Negative	Well	T3N1M0	IVA	NA	0.521388496	B	4.749600094
CCA_TH_7	1	15.2	Fluke-Pos	Thailand	M	61	Perihilar	No	Negative	Negative	Papillary c	T3N0M0	IIIB	2	0.510057096	B	5.599394325
CCA_TH_8	1	8.866666667	Fluke-Pos	Thailand	F	53	Intrahepatic	No	Negative	Negative	Well	T3N0M0	III	2	0.536433588	B	5.426728912
CCA_TH_9	0	12.43333333	Fluke-Pos	Thailand	M	48	Intrahepatic	No	Negative	Negative	Well	T3N0M0	III	NA	0.509453959	B	5.415378921
CCA_TH_10	0	38.93333333	Fluke-Pos	Thailand	F	69	Intrahepatic	No	NA	NA	NA	T3N1M0	IVA	1	0.493381324	B	4.62050332
CCA_TH_11	1	7.6	Fluke-Pos	Thailand	F	79	Intrahepatic	No	NA	NA	Well	T4N0M0	IVA	1	0.542108626	B	5.072671423
CCA_TH_12	1	10.23333333	Fluke-Pos	Thailand	M	52	Perihilar	No	NA	NA	Well	T2aN1M0	IIIB	NA	0.521279555	B	6.919741934
CCA_TH_13	0	34.23333333	Fluke-Pos	Thailand	M	57	Perihilar	No	NA	NA	Papillary c	T3N0M0	IIIA	1	0.460913074	B	5.385161009
CCA_TH_15	1	15.83333333	Fluke-Pos	Thailand	M	37	Perihilar	No	Negative	Negative	Papillary c	T4N1M0	IIIA	2	0.523118284	B	5.513964644
CCA_TH_16	1	4.966666667	Fluke-Pos	Thailand	F	61	Intrahepatic	No	Positive	Negative	Well	T3N0M0	III	NA	0.525557229	B	8.668214139
CCA_TH_17	1	2.133333333	Fluke-Pos	Thailand	M	66	Intrahepatic	No	Negative	Negative	Well	T3N0M0	III	1	0.495608372	B	5.152702647
CCA_TH_18	1	5.233333333	Fluke-Pos	Thailand	M	56	Intrahepatic	No	NA	NA	Well	T4N1M0	IVA	1	0.534069335	B	5.339616988
CCA_TH_19	1	5.9	Fluke-Pos	Thailand	F	65	Intrahepatic	No	NA	NA	Papillary c	T3N0M0	III	1	0.483361003	B	5.037190435
CCA_TH_20	1	18.56666667	Fluke-Pos	Thailand	M	63	Intrahepatic	No	NA	NA	Papillary c	T4N1M0	IVA	1	0.511946584	B	5.673483186
CCA_TH_21	0	75.16666667	Fluke-Pos	Thailand	F	40	Intrahepatic	No	Negative	Negative	Papillary c	T1N0M0	I	2	0.474221998	B	4.758608319
CCA_TH_22	0	69.96666667	Fluke-Pos	Thailand	F	56	Intrahepatic	No	Negative	Negative	Papillary c	T2N0M0	II	1	0.515782216	B	4.795980839
CCA_TH_23	1	58.66666667	Fluke-Pos	Thailand	F	56	Perihilar	No	NA	NA	Papillary c	T1N0M0	I	2	0.504910153	B	4.848459011
CCA_TH_27	1	37.3	Fluke-Pos	Thailand	F	72	Intrahepatic	No	Negative	Negative	Poor	T3N1M0	IVA	4	0.472781816	A	3.662751563
CCA_TH_28	1	31.5	Fluke-Pos	Thailand	F	52	Intrahepatic	No	Negative	Negative	Well	T3N1M0	IVA	3	0.477339841	B	5.617962203
CCA_TH_31	1	5.133333333	Fluke-Pos	Thailand	M	55	Intrahepatic	No	Negative	Negative	Well	T4N0M0	IVA	1	0.552527978	B	7.741719926
CCA_TH_32	1	12.56666667	Fluke-Pos	Thailand	M	50	Perihilar	No	NA	NA	Papillary c	T2bN1M0	IIIB	NA	0.556206949	B	5.809403013
CCA_TH_35	1	0.1	Fluke-Pos	Thailand	M	76	Perihilar	No	NA	NA	Papillary c	T4N1M1	IVB	NA	0.477021473	B	5.190120203
CCA_TH_43	1	18.63333333	Fluke-Pos	Thailand	F	38	Intrahepatic	No	NA	NA	Well	T2bN1M0	IVA	1	0.47126105	B	5.791298769
CCA_TH_44	1	8.533333333	Fluke-Pos	Thailand	M	64	Perihilar	No	NA	NA	Papillary c	T3N0M1	IVB	1	0.514804097	B	6.111418866
CCA_TH_47	0	57.13333333	Fluke-Pos	Thailand	M	66	Perihilar	No	NA	NA	Papillary c	TisN0m0	0	1	0.549683561	B	5.339356267
CCA_TH_50	0	56.03333333	Fluke-Pos	Thailand	M	60	Perihilar	No	NA	NA	Papillary c	T3N0M0	IIIA	1	0.437198812	B	5.615179452
CCA_TH_55	1	2.733333333	Fluke-Pos	Thailand	M	73	Intrahepatic	No	Negative	Negative	Well	T3N0M0	III	1	0.518318821	B	5.991776272
CCA_TH_56	1	26.16666667	Fluke-Pos	Thailand	M	56	Intrahepatic	No	Negative	Negative	Well	T2bN0M0	II	NA	0.485760288	B	5.233185896

CCA_TH_59	1	12.8	Fluke-Pos	Thailand	M	47	Intrahepatic	No	NA	NA	Papillary c	T3N0M0	III	1	0.519681967	B	5.501395266
CCA_TH_65	1	9.266666667	Fluke-Pos	Thailand	M	42	Intrahepatic	No	NA	NA	Papillary c	T3N1M0	IVA	1	0.522635611	B	6.645401788
CCA_TH_69	0	37.03333333	Fluke-Pos	Thailand	F	48	Intrahepatic	No	NA	NA	Papillary c	T2bN0M0	II	4	0.446839009	A	3.940691184
CCA_TH_70	1	3.833333333	Fluke-Pos	Thailand	F	49	Intrahepatic	No	NA	NA	Papillary c	T3N1M0	IVA	NA	0.483336519	B	5.796500864
CCA_TH_72	0	36.36666667	Fluke-Pos	Thailand	M	66	Perihilar	No	NA	NA	Well	T2aN0M0	II	1	0.441958111	B	5.469220293
CCA_TH_75	0	34.56666667	Fluke-Pos	Thailand	M	52	Intrahepatic	No	NA	NA	Papillary c	T3N0M0	III	2	0.496958286	B	5.106416791
CCA_TH_76	1	18.06666667	Fluke-Pos	Thailand	M	73	Intrahepatic	No	NA	NA	Papillary c	T2aN0M0	II	NA	0.531878918	B	5.780245063
CCA_TH_79	1	16.83333333	Fluke-Pos	Thailand	M	64	Intrahepatic	No	NA	NA	Well	T3N1M0	IVA	2	0.532889524	B	5.77500951
CCA_TH_81	1	19.23333333	Fluke-Pos	Thailand	M	49	Perihilar	No	NA	NA	Well	T4N0M1	IVB	1	0.525158515	B	5.712423131
CCA_TH_85	0	30.53333333	Fluke-Pos	Thailand	F	52	Intrahepatic	No	NA	NA	Well	T2aN0M0	II	1	0.542106715	B	5.394211931
CCA_TH_88	1	15.23333333	Fluke-Pos	Thailand	M	64	Intrahepatic	No	NA	NA	Adenosquamo	T3N0M0	III	NA	0.511915648	A	6.591119876
CCA_TH_89	1	3.633333333	Fluke-Pos	Thailand	F	66	Intrahepatic	No	NA	NA	Moderate	T3N1M0	IVA	NA	0.531895339	B	6.540332727
CCA_TH_114	1	8.366666667	Fluke-Pos	Thailand	F	49	Perihilar	No	NA	NA	Well	T2bN1M0	IIIB	NA	0.521895474	B	6.249210137
CCA_TH_121	1	7	Fluke-Pos	Thailand	M	70	Intrahepatic	No	Negative	Negative	Well	T4NXM0	IVA	1	0.5299891	B	6.931190573
CCA_TH_122	1	6	Fluke-Pos	Thailand	M	50	Intrahepatic	No	Negative	Negative	Papillary c	T3N1M0	IVA	1	0.498087724	B	6.727102245
CCA_TH_127	1	20.3	Fluke-Pos	Thailand	F	51	Perihilar	No	Negative	Negative	Well	T4N0M0	IVA	1	0.516129126	B	6.126958845
CCA_TH_128	1	11.33333333	Fluke-Pos	Thailand	M	55	Perihilar	No	Negative	Negative	Well	T4N0M0	IVA	1	0.50075119	B	7.241099526
CCA_TH_130	NA	NA	Fluke-Pos	Thailand	M	51	Perihilar	No	NA	NA	Papillary c	NA	NA	1	0.517471409	B	4.709284591

Supplementary Table-S3: Predicted applicable or not applicable drugs for patients with high estrogen response			
ID	Name	Description	Score
BRD-A15079084	phorbol-12-myristate-13-acetate	PKC activator	99.15
BRD-K92991072	PAC-1	Caspase activator	98.34
BRD-K35687265	ON-01910	PLK inhibitor	97.74
BRD-K80431395	triciribine	AKT inhibitor	97.72
BRD-K86003836	flubendazole	Tubulin inhibitor	97.45
BRD-K99498722	NPI-2358	Tubulin inhibitor	96.71
BRD-K25504083	cytochalasin-d	Actin polymerization inhibitor	96.65
BRD-U44700465	HG-5-88-01	Protein kinase inhibitor	96.17
BRD-K21350491	phenamil	TRPV antagonist	96.15
BRD-K26997899	SA-792574	Microtubule inhibitor	95.53
BRD-K57011718	UK-356618	Metalloproteinase inhibitor	95.45
BRD-K12539581	nocodazole	Tubulin inhibitor	95.42
BRD-K72895815	SSR-69071	Leukocyte elastase inhibitor	95.4
BRD-K91145395	prostratin	PKC activator	95.39
BRD-A52650764	ingenol	PKC activator	95.07
BRD-K28360340	TW-37	BCL inhibitor	94.58
BRD-K91623615	ABT-751	Tubulin inhibitor	92.93
BRD-K94325918	kinetin-riboside	Apoptosis stimulant	91.92
BRD-A28105619	cucurbitacin-i	JAK inhibitor	91.83
BRD-K37865504	LY-2183240	FAAH inhibitor	91.45
BRD-K55420858	mirin	MRE11A exonuclease inhibitor	90.85
BRD-K77987382	mebendazole	Tubulin inhibitor	90.29
BRD-K62012036	acitretin	Retinoid receptor agonist	90.11
BRD-A54927599	KF-38789	P-selectin inhibitor	88.96
BRD-K22096725	ALW-II-49-7	Ephrin inhibitor	88.79
BRD-K28120222	parthenolide	NFkB pathway inhibitor	85.85
BRD-K02526760	QS-11	ARFGAP inhibitor	85.63
BRD-K17140735	SCH-79797	Proteasome inhibitor	85.19
BRD-K90382497	GW-843682X	PLK inhibitor	85.06
BRD-K96778649	tyrphostin-47	EGFR inhibitor	84.81
BRD-K47869605	podophyllotoxin	Microtubule inhibitor	84.79
BRD-K89152108	liothyronine	Thyroid hormone stimulant	84.61
BRD-K19894101	MST-312	Telomerase inhibitor	84.42
BRD-K52075715	oxibendazole	Tubulin inhibitor	83.34
BRD-A48570745	ivermectin	GABA receptor agonist	83.24
BRD-K19554809	MK-212	Serotonin receptor agonist	83.24
BRD-A33833419	TER-14687	Inhibitor of translocation of PKCq in T cells	82.9
BRD-M30523314	vinorelbine	Tubulin inhibitor	82.68
BRD-A55594068	vinblastine	Microtubule inhibitor	81.7
BRD-K66296774	fluvastatin	HMGCR inhibitor	81
BRD-K62200014	anagrelide	Phosphodiesterase inhibitor	80.99
BRD-K76907295	VU-0418947-2	HIF modulator	80.7
BRD-K15402119	huperzine-a	Acetylcholinesterase inhibitor	80.39
BRD-A87387433	cefpodoxime	Bacterial cell wall synthesis inhibitor	80.28
BRD-K67298865	SB-431542	TGF beta receptor inhibitor	80.22
BRD-K47693913	evoxine	Furoquinoline alkaloid	79.74
BRD-K86727142	embelin	HCV inhibitor	78.61
BRD-K02965346	SU-11274	Hepatocyte growth factor receptor inhibitor	77.75
BRD-K37456065	VU-0365114-2	M5 modulator	77.58
BRD-K38449220	seneciphylline	Cytochrome P450 inhibitor	77.53
BRD-A50157456	terbutaline	Adrenergic receptor agonist	77.52
BRD-K94270326	ecopipam	Dopamine receptor antagonist	77.17
BRD-K89162000	tandutinib	FLT3 inhibitor	76.93
BRD-K58772419	AZD-6482	PI3K inhibitor	76.29
BRD-K81062487	taurocholic-acid	Bile acid	76.04
BRD-K56614220	clofazimine	GK0582 inhibitor	75.78
BRD-K18678457	ZD-7288	HCN channel blocker	74.09
BRD-K82823804	SA-792987	PKC inhibitor	73.38
BRD-K29733039	deforolimus	MTOR inhibitor	72.04
BRD-K14550461	doxercalciferol	Vitamin D receptor agonist	71.89
BRD-K96527333	dehydroisoandosterone	GABA receptor modulator	71.66
BRD-A53576514	orphenadrine	Acetylcholine receptor antagonist	71.42
BRD-K83289131	CAY-10618	NAMPT inhibitor	70.34
BRD-K59419204	AM-281	Cannabinoid receptor antagonist	70.27
BRD-K44432556	VU-0418946-1	HIF modulator	70.08
BRD-K40213712	SAL-1	Adenosine receptor antagonist	69.55
BRD-K54472332	elvitegravir	HIV integrase inhibitor	69.36

BRD-K22631935	neurodazine	Neurogenesis of non-pluripotent C2C12 myoblast inducer	68.94
BRD-K79437791	acetyl-farnesyl-cysteine	Inhibitor of methylation of endogenous isoprenylated proteins	68.79
BRD-K89930444	AG-592	Tyrosine kinase inhibitor	68.64
BRD-A85472596	L-670596	Prostanoid receptor antagonist	68.12
BRD-K61401890	deguelin	NADH-ubiquinone oxidoreductase (Complex I) inhibitor	67.84
BRD-K72264770	QW-BI-011	Histone lysine methyltransferase inhibitor	67.63
BRD-K37194137	III606050	Cytochrome P450 inhibitor	67.51
BRD-A45498368	WYE-125132	MTOR inhibitor	67.15
BRD-K59753975	vindesine	Tubulin inhibitor	66.86
BRD-K87919739	tyrphostin-AG-825	Receptor tyrosine protein kinase inhibitor	66.54
BRD-K40175214	torin-1	MTOR inhibitor	66.42
BRD-A08003242	rhodomyrtoxin-b	sodium fluorescein uptake inhibitor	66.29
BRD-K19796430	erismodegib	Smoothened receptor antagonist	66
BRD-K74430258	1,2-dichlorobenzene	Hepatotoxicant	65.64
BRD-K07507905	BRL-37344	Adrenergic receptor agonist	65.64
BRD-A16665823	butoconazole	Bacterial cell wall synthesis inhibitor	64.81
BRD-A26032986	zaldaride	Calmodulin antagonist	64.8
BRD-A45333398	periplocymarin	Apoptosis stimulant	64.79
BRD-K26979635	NS-3694	Glutamate receptor antagonist	64.79
BRD-K52751261	TAK-715	p38 MAPK inhibitor	64.54
BRD-K74402642	NSC-632839	Ubiquitin specific protease inhibitor	64.22
BRD-K66792149	quinoclamine	Algicide	64.22
BRD-K15616905	CCCP	Mitochondrial oxidative phosphorylation uncoupler	64.2
BRD-K76698671	HNHA	HDAC inhibitor	64.13
BRD-K68867920	quetiapine	Dopamine receptor antagonist	63.7
BRD-K92731339	perindopril	ACE inhibitor	63.43
BRD-A10420615	cyclopiazonic-acid	ATPase inhibitor	63.11
BRD-K54708045	nTZDpa	PPAR receptor agonist	62.99
BRD-K10705233	GW-405833	Cannabinoid receptor agonist	62.66
BRD-K13087974	4,5-dianilinophthalimide	EGFR inhibitor	61.94
BRD-A01317026	7,8-dihydro-L-biopterin	Dihydroneopterin aldolase inhibitor	61.52
BRD-K82109576	vincristine	Tubulin inhibitor	61.16
BRD-A69636825	diltiazem	Calcium channel blocker	61.14
BRD-K93433262	alfacalcidol	Vitamin D receptor agonist	61.02
BRD-K81376179	TCS-359	FLT3 inhibitor	60.43
BRD-A17819071	gedunin	HSP inhibitor	60.11
BRD-K59637651	NSC-119889	Protein synthesis inhibitor	60.07
BRD-K86465814	HO-013	PPAR receptor agonist	60.03
BRD-K16406336	methylene-blue	Guanylyl cyclase inhibitor	59.81
BRD-K60476892	YC-1	Guanylyl cyclase activator	59.57
BRD-K56450366	NSC-94258	Antineoplastic	59.29
BRD-K96720755	relcovaptan	Vasopressin receptor antagonist	59.22
BRD-K83837640	JNJ-26854165	HDAC inhibitor	59.13
BRD-K85402309	dovitinib	EGFR inhibitor	58.87
BRD-K92000912	AM-251	Cannabinoid receptor antagonist	58.52
BRD-K54095730	CMPD-1	p38 MAPK inhibitor	58.04
BRD-K11911061	GR-127935	Serotonin receptor antagonist	57.8
BRD-A79314293	cephalosporanic-acid	Bacterial cell wall synthesis inhibitor	57.6
BRD-A72703248	SKF-96365	Calcium channel blocker	57.51
BRD-K28667793	pyrazinamide	Fatty acid synthase inhibitor	56.98
BRD-K74305673	IKK-2-inhibitor-V	IKK inhibitor	56.85
BRD-K08109516	L-701324	Glutamate receptor antagonist	56.5
BRD-K42098891	protriptyline	Tricyclic antidepressant	56.12
BRD-K02113016	olaparib	PARP inhibitor	55.76
BRD-K09255212	clioquinol	Chelating agent	55.76
BRD-K06878038	deferiprone	Chelating agent	55.12
BRD-A51820102	econazole	Bacterial cell wall synthesis inhibitor	55.05
BRD-K69328504	L-690488	Inositol monophosphatase inhibitor	54.66
BRD-K55034111	pefloxacin	Bacterial DNA gyrase inhibitor	54.52
BRD-K05977823	tenovins	SIRT inhibitor	54.31
BRD-M86331534	pyrvinium-pamoate	AKT inhibitor	54.08
BRD-K78278890	NM-PP1	Mutant kinase inhibitor	54.01
BRD-K08219523	5-nonyloxytryptamine	Serotonin receptor agonist	53.73
BRD-K43764301	dexketoprofen	Cyclooxygenase inhibitor	53.57
BRD-K13049116	BMS-754807	IGF-1 inhibitor	53.09
BRD-K92301463	16,16-dimethylprostaglandin-e2	Prostanoid receptor agonist	53.01
BRD-K39120595	bithionol	Autotaxin inhibitor	52.86
BRD-A34205397	suloctidil	Adrenergic receptor antagonist	52.75
BRD-K67013324	luzindole	Melatonin receptor antagonist	52.61
BRD-K11801786	trimidox	Ribonucleotide reductase inhibitor	52.47
BRD-K50938287	sumatriptan	Serotonin receptor agonist	52.45

BRD-K98490050	amsacrine	Topoisomerase inhibitor	52.11
BRD-K26801045	pipamperone	Dopamine receptor antagonist	51.73
BRD-K20995441	U-54494A	Opioid receptor agonist	51.31
BRD-A00267231	hemado	Adenosine receptor agonist	51.12
BRD-A63998256	helveticoside	ATPase inhibitor	51.06
BRD-A39522003	OMDM-2	FAAH inhibitor	50.51
BRD-K84266862	BRL-50481	Phosphodiesterase inhibitor	50.23
BRD-A25143711	hydrocortisone	Glucocorticoid receptor agonist	-50.24
BRD-K95921201	reserpine	Vesicular monoamine transporter inhibitor	-50.25
BRD-K12260308	xanthoxyline	Antifungal	-50.74
BRD-K48923948	BMS-641988	Androgen receptor antagonist	-50.88
BRD-A63836183	PD-123319	Angiotensin receptor antagonist	-50.97
BRD-K50388907	fenofibrate	PPAR receptor agonist	-51.16
BRD-K73391359	quinisocaine	Local anesthetic	-51.42
BRD-K82562631	tolmetin	Cyclooxygenase inhibitor	-51.5
BRD-A55756846	H-7	PKA inhibitor	-51.55
BRD-K95763993	trapidil	PDGFR receptor inhibitor	-51.67
BRD-K53561341	KIN001-220	Aurora kinase inhibitor	-52.17
BRD-A56020723	CA-074-Me	Cathepsin inhibitor	-52.37
BRD-K34154330	tracazolate	GABA receptor modulator	-52.63
BRD-K53123955	niridazole	Phosphofructokinase inhibitor	-52.73
BRD-K43389675	daunorubicin	RNA synthesis inhibitor	-52.94
BRD-A02333338	cyclopamine	Smoothened receptor antagonist	-52.94
BRD-K86434416	selegiline	Monoamine oxidase inhibitor	-52.96
BRD-A53952395	prilocaine	Local anesthetic	-53.23
BRD-K28428262	brivanib	FGFR inhibitor	-53.27
BRD-K01436366	XMD-1150	Leucine rich repeat kinase inhibitor	-53.6
BRD-K64755930	etazolate	Phosphodiesterase inhibitor	-53.61
BRD-K98493452	honokiol	AKT inhibitor	-53.63
BRD-A44090213	indoprofen	Cyclooxygenase inhibitor	-53.74
BRD-K57718010	pentylenetetrazol	GABA receptor antagonist	-53.86
BRD-K76810206	nicergoline	Adrenergic receptor antagonist	-53.92
BRD-K93325701	damnacanthal	SRC inhibitor	-54.03
BRD-K92726801	hydrastinine	Haemostatic agent	-54.08
BRD-K41160163	fenobam	Glutamate receptor antagonist	-54.27
BRD-A53077924	tianeptine	Selective serotonin reuptake enhancer (SSRE)	-54.48
BRD-K06234293	LY-364947	TGF beta receptor inhibitor	-54.68
BRD-A65597028	RX-821002	Adrenergic receptor antagonist	-54.85
BRD-K85871428	SC-68376	p38 MAPK inhibitor	-54.88
BRD-K49328571	dasatinib	BCR-ABL kinase inhibitor	-54.91
BRD-K83010055	VU-0415374-1	Glutamate receptor modulator	-54.94
BRD-K89708791	rifaximin	RNA synthesis inhibitor	-55.05
BRD-A06352508	SB-218078	CHK inhibitor	-55.54
BRD-K72093121	vidarabine	Antiviral	-55.62
BRD-A59174698	ritodrine	Adrenergic receptor agonist	-55.91
BRD-K56700933	phenethyl-isothiocyanate	Antineoplastic	-55.93
BRD-K05464208	JX-401	p38 MAPK inhibitor	-56.01
BRD-A61858259	CAY-10415	Insulin sensitizer	-56.19
BRD-K69650333	idarubicin	Topoisomerase inhibitor	-56.89
BRD-A52588987	SKF-83566	Dopamine receptor antagonist	-56.92
BRD-K89014967	AS-703026	MEK inhibitor	-57.14
BRD-K23875128	RHO-kinase-inhibitor-III[rockout]	Rho associated kinase inhibitor	-57.15
BRD-A72441487	stiripentol	GABA uptake inhibitor	-57.24
BRD-K05926469	lenalidomide	Antineoplastic	-57.31
BRD-K93095519	SJ-172550	MDM inhibitor	-57.5
BRD-K99451608	lopinavir	HIV protease inhibitor	-57.75
BRD-K63550407	erythromycin	NFkB pathway inhibitor	-57.75
BRD-A37837077	cyclazosin	Adrenergic receptor antagonist	-57.96
BRD-K13390322	AT-7519	CDK inhibitor	-58.48
BRD-K07691486	roscovitine	CDK inhibitor	-58.51
BRD-A92177080	betamethasone	Glucocorticoid receptor agonist	-58.89
BRD-A62035778	scopolamine	Acetylcholine receptor antagonist	-59
BRD-K76674262	homoharringtonine	Protein synthesis inhibitor	-59.48
BRD-K67174588	toremifene	Estrogen receptor antagonist	-60.61
BRD-K57080016	selumetinib	MEK inhibitor	-60.65
BRD-A73368467	fexofenadine	Histamine receptor antagonist	-60.9
BRD-A73909368	dactinomycin	RNA polymerase inhibitor	-61.2
BRD-K97056771	HY-11007	BCR-ABL kinase inhibitor	-61.44
BRD-K18816859	L-694247	Serotonin receptor agonist	-61.56
BRD-K23204545	busulfan	DNA inhibitor	-62.23
BRD-A67438293	treprostinil	Prostacyclin analog	-62.88

BRD-K39983086	loteprednol	Glucocorticoid receptor agonist	-63.39
BRD-K75532464	FTI-276	Farnesyltransferase inhibitor	-63.45
BRD-K45988865	tetramethylsilane	Internal standard for NMR spectroscopy	-63.67
BRD-K18787491	U-0126	MEK inhibitor	-63.7
BRD-A78322124	dobutamine	Adrenergic receptor agonist	-64.48
BRD-K25906698	immepip	Histamine receptor agonist	-64.96
BRD-M16762496	PIK-75	DNA protein kinase inhibitor	-65.02
BRD-A07824748	flavanone	11-beta-HSD1 inhibitor	-65.25
BRD-K70914287	BIBX-1382	EGFR inhibitor	-65.43
BRD-K62374253	rufloxacin	Bacterial DNA gyrase inhibitor	-65.56
BRD-K43164539	cholic-acid	Bile acid	-65.62
BRD-K77133231	PD-169316	p38 MAPK inhibitor	-66.08
BRD-K96037667	norethindrone	Progesterone receptor agonist	-66.32
BRD-K96119599	leucodin	Melanin inhibitor	-66.5
BRD-K08996725	zolantidine	Histamine receptor antagonist	-66.95
BRD-K13810148	givinostat	HDAC inhibitor	-67.44
BRD-K83794624	pirarubicin	Topoisomerase inhibitor	-67.77
BRD-K48935217	epothilone	Microtubule inhibitor	-68.58
BRD-A16934955	nalbuphine	Opioid receptor agonist	-68.6
BRD-K41445866	alfaxalone	Chloride channel agonist	-68.88
BRD-K06895174	cisapride	Serotonin receptor agonist	-69.3
BRD-K81847782	scandenin	Plant compound with antimicrobial activity	-69.51
BRD-K17953061	staurosporine	PKC inhibitor	-69.76
BRD-K04548931	pidorubicine	Topoisomerase inhibitor	-70.14
BRD-K41859756	NVP-AUY922	HSP inhibitor	-70.39
BRD-A76279427	myriocin	Serine palmitoyltransferase inhibitor	-70.58
BRD-K65417056	meprylcaine	Local anesthetic	-70.72
BRD-A80638690	floxuridine	DNA synthesis inhibitor	-71.07
BRD-K50387473	XMD-892	MAP kinase inhibitor	-71.34
BRD-K32584078	BML-257	AKT inhibitor	-71.45
BRD-K52620403	STO-609	Calmodulin antagonist	-71.64
BRD-K95885906	quercetagetin	PIM inhibitor	-71.84
BRD-K74236984	UNC-0321	Histone lysine methyltransferase inhibitor	-72.49
BRD-K01555864	dibenzoylmethane	Antineoplastic	-72.64
BRD-A47706533	L-BSO	Glutathione transferase inhibitor	-72.92
BRD-K89375097	pirenzepine	Acetylcholine receptor antagonist	-73.31
BRD-K87909389	alvocidib	CDK inhibitor	-73.81
BRD-K64052750	gefitinib	EGFR inhibitor	-74.73
BRD-K51476772	ST-638	Tyrosine kinase inhibitor	-74.74
BRD-K33459542	ditolylguanidine	Sigma receptor agonist	-75.32
BRD-K06543683	bisindolylmaleimide-ix	CDK inhibitor	-75.46
BRD-K49865102	PD-0325901	MEK inhibitor	-77.37
BRD-K73319509	PF-04217903	c-Met inhibitor	-78.03
BRD-A41722204	sulmazole	Adenosine receptor antagonist	-78.54
BRD-K99696746	fatostatin	SREBP inhibitor	-78.62
BRD-A01787639	naftopidil	Adrenergic receptor antagonist	-80.03
BRD-A13122391	triptolide	RNA polymerase inhibitor	-80.37
BRD-A43671941	oxprenolol	Adrenergic receptor antagonist	-80.57
BRD-U44618005	WH-4023	SRC inhibitor	-80.89
BRD-K92093830	doxorubicin	Topoisomerase inhibitor	-81.63
BRD-K24576554	AT-9283	JAK inhibitor	-83.69
BRD-K53414658	tivozanib	VEGFR inhibitor	-83.78
BRD-K96740444	itopride	Dopamine receptor antagonist	-83.97
BRD-A45543382	metrizamide	Radiopaque medium	-84.08
BRD-K08924299	palonosetron	Serotonin receptor antagonist	-84.16
BRD-K12867552	THM-I-94	HDAC inhibitor	-84.45
BRD-K15519488	CS-110266	Dopamine receptor agonist	-85.02
BRD-K81418486	vorinostat	HDAC inhibitor	-86.43
BRD-K50018155	RS-67506	Serotonin receptor partial agonist	-87.61
BRD-A39255369	DCPIB	Chloride channel blocker	-88.18
BRD-K62810658	PD-98059	MEK inhibitor	-88.32
BRD-K85985071	ellipticine	Topoisomerase inhibitor	-88.51
BRD-A60197193	amisulpride	Dopamine receptor antagonist	-88.95
BRD-K69840642	ISOX	HDAC inhibitor	-89.14
BRD-K48722258	dilazep	Adenosine reuptake inhibitor	-89.24
BRD-K40742111	baeomycesic-acid	Lipoxygenase inhibitor	-89.96
BRD-K79090631	CGP-60474	CDK inhibitor	-90.31
BRD-K58299615	RO-90-7501	Beta amyloid inhibitor	-90.68
BRD-A25687296	emetine	Protein synthesis inhibitor	-90.94
BRD-K49456190	prima-1-met	thioredoxin inhibitor	-91.05
BRD-K12502280	TG-101348	FLT3 inhibitor	-91.19

BRD-K64935403	ebelactone-b	Lipase inhibitor	-91.27
BRD-K41895714	AS-605240	PI3K inhibitor	-91.27
BRD-A39646320	HC-toxin	HDAC inhibitor	-91.35
BRD-K22503835	scriptaid	HDAC inhibitor	-91.65
BRD-K05104363	PD-184352	MEK inhibitor	-92.67
BRD-K36529613	PU-H71	HSP inhibitor	-93.59
BRD-K94176593	TWS-119	Glycogen synthase kinase inhibitor	-93.75
BRD-K64606589	apicidin	HDAC inhibitor	-94.06
BRD-K23192422	lestaurtinib	FLT3 inhibitor	-94.12
BRD-K21565985	xylazine	Adrenergic receptor agonist	-95.03
BRD-K02130563	panobinostat	HDAC inhibitor	-95.17
BRD-K14807180	SB-221284	Serotonin receptor antagonist	-95.48
BRD-A29731977	17-hydroxyprogesterone-caproate	progesterone receptor agonist	-97.35
BRD-U51951544	ZG-10	JNK inhibitor	-97.39
BRD-K37814297	acepromazine	Dopamine receptor antagonist	-98.2
BRD-K54330070	SB-202190	p38 MAPK inhibitor	-98.86

Supplementary Table-S4: The correlation between the IC50 of drugs and the GSVA score of estrogen response in cancer cell lines from GDSC

Drugs	Spearman R	p value	Number of cell lines	Target	Targeted patheays
TL-1-85	0.492079999	0	863	TAK	Other, kinases
GSK429286A	0.477843699	0	862	ROCK1, ROCK2	Cytoskeleton
KIN001-260	0.465944458	0	862	IKKB	Other, kinases
KIN001-270	0.440437106	0	863	CDK9	Cell cycle
UNC0638	0.439932915	0	867	G9a and GLP methyltransferases	Chromatin histone methylation
NG-25	0.438839607	0	863	TAK1, MAP4K2	Other, kinases
XMD14-99	0.436504561	0	864	ALK, CDK7, LTK, others	Other, kinases
Masitinib	0.435465002	0	863	KIT, PDGFRA, PDGFRB	RTK signaling
QL-XI-92	0.434400822	0	864	DDR1	Cytoskeleton
BX-912	0.433379688	0	863	PDK1 (PDPK1)	Metabolism
BIX02189	0.431341094	0	862	MEK5, ERK5	ERK MAPK signaling
kb NB 142-70	0.426726964	0	868	PKD	Other, kinases
Y-39983	0.419675771	0	862	ROCK	Cytoskeleton
Tivozanib	0.418705827	0	862	VEGFR1, VEGFR2, VEGFR3	RTK signaling
CAY10603	0.41471858	0	855	HDAC1, HDAC6	Chromatin histone acetylation
JW-7-24-1	0.414256421	0	864	LCK	Other, kinases
THZ-2-102-1	0.412465992	0	841	CDK7	Cell cycle
NPK76-II-72-1	0.412021569	0	862	PLK3	Cell cycle
Tubastatin A	0.409580199	0	861	HDAC1, HDAC6, HDAC8	Chromatin histone acetylation
Daporinad	0.408989804	0	843	NAMPT	Metabolism
T0901317	0.404754866	0	857	LXR, FXR	Other
KIN001-244	0.401032996	0	862	PDK1 (PDPK1)	Metabolism
KIN001-236	0.399594337	0	863	Angiopoietin-1 receptor	RTK signaling
I-BET-762	0.397565826	0	860	BRD2, BRD3, BRD4	Chromatin other
MIM1	0.397274427	0	853	MCL-1	Apoptosis regulation
Zibotentan	0.397044508	0	863	Endothelin-1 receptor (EDNRA)	Other
AT7867	0.39653711	0	868	AKT	PI3K/MTOR signaling
PIK-93	0.392930662	0	862	PI3Kgamma	PI3K/MTOR signaling
Selisistat	0.392602283	0	860	SIRT1	Chromatin histone acetylation
STF-62247	0.392307779	0	861	Autophagy inducer	Other
Fedratinib	0.39095538	0	864	JAK2	Other, kinases
YM201636	0.389265075	0	863	PIKFYVE	PI3K/MTOR signaling
NVP-BHG712	0.38852131	0	863	EPHB4	RTK signaling
VX-702	0.387818162	0	889	p38	JNK and p38 signaling
TPCA-1	0.386237662	0	863	IKK2	Other, kinases
XMD15-27	0.384750344	0	864	CAMK2	Other, kinases
IMD-0354	0.38442774	0	861	IKKb	Other, kinases
Quizartinib	0.384353694	0	864	FLT3	RTK signaling
Tenovin-6	0.383776885	0	868	SIRT	Chromatin histone acetylation
Methotrexate	0.382218911	0	894	Antimetabolite	DNA replication
AZD4547	0.382053922	0	865	FGFR1, FGFR2, FGFR3	RTK signaling
PI-103	0.381861994	0	851	PI3Kalpha, DAPK3, CLK4, PIM3, HIPK2	Other, kinases
UNC1215	0.381726777	0	859	L3MBTL3	Chromatin other
XMD13-2	0.38153805	0	863	RIPK1	Apoptosis regulation
TL-2-105	0.380995687	0	864	not defined	Other
Lestaurtinib	0.37307501	0	892	FLT3, JAK2, NTRK1, NTRK2, NTRK3	Other, kinases
Vorinostat	0.372816382	0	895	HDAC inhibitor Class I, IIa, IIb, IV	Chromatin histone acetylation
Brivanib, BMS-540215	0.372627453	0	867	VEGFR, PDGFR	RTK signaling
Ruxolitinib	0.369491464	0	865	JAK1, JAK2	Other, kinases
Venotoclax	0.369289265	0	857	BCL-2 selective	Apoptosis regulation
VNLG/124	0.368334213	0	859	HDAC,RAR	Chromatin histone acetylation
GSK319347A	0.368182739	9.68E-14	383	IKK	Other, kinases
eEF2K Inhibitor, A-484954	0.367996039	0	865	eEF2K	Other, kinases
FEN1 3940	0.36711945	0	876	FEN1	Genome integrity
AR-42	0.364194188	0	850	HDAC1	Chromatin histone acetylation
UNC0642	0.361277837	0	844	G9a(EHMT2), GLP(EHMT1)	Chromatin histone methylation
OSI-027	0.360291776	0	851	MTORC1, MTORC2	PI3K/MTOR signaling
PD173074	0.358174442	0	895	FGFR1, FGFR2, FGFR3	RTK signaling
Sphingosine Kinase 1 Inhibitor II	0.356955409	0	868	Sphingosine Kinase	Other, kinases
OSI-930	0.354520001	0	863	KIT	RTK signaling
SNX-2112	0.354326969	0	853	HSP90	Protein stability and degradation
ACY-1215	0.35385509	0	866	HDAC6	Chromatin histone acetylation
Crizotinib	0.352592734	9.65E-13	386	MET, ALK, ROS1	RTK signaling
Temozolomide	0.350153128	0	858	DNA alkylating agent	DNA replication
Nilotinib	0.348963856	0	894	ABL	ABL signaling
GSK269962A	0.348864063	0	626	ROCK1, ROCK2	Cytoskeleton
IPA-3	0.346863847	0	821	PAK1	Cytoskeleton
QL-X-138	0.346706122	0	852	BTK	Other, kinases
CX-5461	0.346208906	0	857	RNA Polymerase 1	Other
WYE-125132	0.345490442	0	856	mTOR	PI3K/MTOR signaling
GSK1070916	0.344408013	0	818	AURKA, AURKC	Mitosis
LDN-193189	0.34383934	0	859	BMP	Other
Imatinib	0.343328948	3.80E-12	387	ABL, KIT, PDGFR	Other, kinases
QL-XII-61	0.342371784	1.72E-13	438	BMX, BTK	Other, kinases
Nutlin-3a (-)	0.341826039	0	896	MDM2	p53 pathway
AZD7969	0.34059951	0	879	GSK3B	WNT signaling
Vismodegib	0.340351334	0	896	SMO	Other
AZD6094	0.337962251	0	881	MET	RTK signaling
CD532	0.337829747	0	869	AURKA	Mitosis

ETP-45835	0.336233981	0	868	MNK1, MNK2	Other, kinases
MCT1_6447	0.335707177	0	863	MCT1	Cell cycle
Belinostat	0.334163371	0	838	HDAC1	Chromatin histone acetylation
Axitinib	0.332471989	0	888	PDGFR, KIT, VEGFR	RTK signaling
CP466722	0.331769283	0	864	ATM	Genome integrity
Genentech Cpd 10	0.329378049	0	862	AURKA, AURKB	Mitosis
SGC0946	0.329305036	0	858	DOT1L	Chromatin histone methylation
SN-38	0.328391907	0	887	TOP1	DNA replication
ZG-10	0.327657808	6.36E-13	458	JNK1	JNK and p38 signaling
Flavopiridol	0.327646526	0	855	CDK	Cell cycle
Torin 2	0.32741328	0	823	mTOR	PI3K/MTOR signaling
PLX-4720	0.325916872	0	884	BRAF	ERK MAPK signaling
PFI-3	0.32577309	0	804	SMARCA2, SMARCA4, PB1	Chromatin other
JAK1_3715	0.325406427	0	882	JAK1	Other, kinases
BAY-61-3606	0.324028297	0	823	SYK	Other, kinases
Cyclopamine	0.323023884	1.25E-10	378	SMO	Other
BX795	0.322388499	0	895	TBK1, PDK1 (PDPK1), IKK, AURKB, AURKC	Other, kinases
BMS-345541	0.319328062	0	865	IKK1, IKK2	Other, kinases
PHA-793887	0.317741196	0	862	CDK2, CDK7, CDK5	Cell cycle
MPS-1-IN-1	0.317634426	0	861	MPS1	Mitosis
Foretinib	0.317085132	0	860	MET, KDR, TIE2, VEGFR3/FLT4, RON, PDGFR, FGFR1, EGFR	RTK signaling
Tamoxifen	0.314384644	0	874	ESR1	Hormone-related
KIN001-266	0.314330458	0	862	MAP3K8	ERK MAPK signaling
IC-87114	0.313445516	0	862	PI3Kdelta	PI3K/MTOR signaling
Ara-G	0.313398915	0	857	Anti-metabolite	Other
Parthenolide	0.310321988	5.39E-10	383	HDAC1	Chromatin histone acetylation
C-75	0.308137935	0	859	FAS	Apoptosis regulation
NSC-207895	0.307620479	0	859	MDM4	p53 pathway
SB590885	0.307613691	0	861	BRAF	ERK MAPK signaling
SU11274	0.307299495	0	867	MET	RTK signaling
ARRY-520	0.307171951	0	851	KIF11	Mitosis
Olaparib	0.306185867	0	856	PARP1, PARP2	Genome integrity
Ispinesib Mesylate	0.303948441	0	860	KSP	Mitosis
Panobinostat	0.301907931	0	849	HDAC	Chromatin histone acetylation
AZD7762	0.30085065	0	888	CHEK1, CHEK2	Cell cycle
THZ-2-49	0.298656697	0	850	CDK9	Cell cycle
SB52334	0.298452615	0	860	ALK5	Other, kinases
Linifanib	0.297732682	0	864	VEGFR1, VEGFR2, VEGFR3, CSF1R, FLT3, KIT	RTK signaling
Mirin	0.297578394	0	817	MRE11	Genome integrity
Pemetrexed	0.29658352	0	850	TYMS	DNA replication
NSC319726	0.296160346	0	827	P53 Mut specific	p53 pathway
NU7441	0.295171388	0	889	DNAPK	Genome integrity
LIMK1 inhibitor BMS4	0.294756116	0	868	LIMK1	Cytoskeleton
Dabrafenib	0.294181103	0	823	BRAF	ERK MAPK signaling
Lenalidomide	0.291555133	0	894	CRBN	Protein stability and degradation
Veliparib	0.288607175	0	895	PARP1, PARP2	Genome integrity
Idelalisib	0.288558692	0	865	PI3Kdelta	PI3K/MTOR signaling
XMD11-85h	0.288180191	3.29E-10	458	BRSK2, FLT4, MARK4, PRKCD, RET, SRPK1	Other, kinases
FMK	0.286088846	1.33E-15	751	RSK	Other, kinases
PHA-665752	0.285662082	1.06E-08	387	MET	RTK signaling
Tretinoin	0.285624597	0	884	Retinoic acid	Other
CHIR-99021	0.285596176	0	811	GSK3A, GSK3B	WNT signaling
Voxtalisib	0.284732554	0	868	PI3K (class 1), DNAPK, MTOR	PI3K/MTOR signaling
CUDC-101	0.283440026	0	844	HDAC1-10, EGFR, ERBB2	Other
Ponatinib	0.283130382	0	828	ABL, PDGFRA, VEGFR2, FGFR1, SRC, TIE2, FLT3	Other, kinases
WZ3105	0.28293112	0	864	SRC, ROCK2, NTRK2, FLT3, IRAK1, others	Other
SL0101	0.280663997	0	881	RSK, AURKB, PIM1, PIM3	Other, kinases
Cabozantinib	0.278815068	0	862	VEGFR, MET, RET, KIT, FLT1, FLT3, FLT4, TIE2,AXL	RTK signaling
GW-2580	0.278678543	0	864	CSF1R	RTK signaling
AGI-6780	0.274991934	2.22E-16	860	IDH2(R140Q)	Metabolism
AZD8055	0.27333621	0	885	MTORC1, MTORC2	PI3K/MTOR signaling
TAK-715	0.272034161	4.44E-16	865	p38alpha, p38beta	JNK and p38 signaling
Alisertib	0.271036437	1.11E-15	843	AURKA	Mitosis
XMD8-85	0.266890248	1.38E-07	378	ERK5, BET	Other
Amuvatinib	0.266729636	2.00E-15	857	KIT, PDGFRA, FLT3	RTK signaling
PAC-1	0.26605966	1.31E-14	811	Procaspase-3, Procaspase-7	Apoptosis regulation
Navitoclax	0.264663871	8.88E-16	891	BCL2, BCL-XL, BCL-W	Apoptosis regulation
RU-SKI 43	0.257375297	1.82E-14	859	Shh	Other
SB216763	0.256688619	1.22E-13	809	GSK3A, GSK3B	WNT signaling
Salubrinal	0.255564152	4.59E-07	379	EIF2A	Other
Alectinib	0.253629546	4.17E-14	861	ALK	RTK signaling
Temsirolimus	0.253514196	2.35E-14	879	MTOR	PI3K/MTOR signaling
XMD8-92	0.253202019	3.80E-08	459	MAPK7	ERK MAPK signaling
5-Fluorouracil	0.252575799	5.93E-14	858	Antimetabolite (DNA & RNA)	Other
IOX2	0.250273718	5.62E-14	876	EGLN1	Other
Bicalutamide	0.250150669	1.95E-14	886	AR	Hormone-related
CAY10566	0.249416133	1.37E-13	855	Stearoyl-CoA desaturase	Other
GSK1059615	0.249215558	1.43E-13	855	PI3K	PI3K/MTOR signaling
QL-XII-47	0.249139762	1.51E-13	854	BTK, BMX	Other, kinases
PFI-1	0.248604129	8.06E-14	877	BRD4	Chromatin other
SB505124	0.244525382	6.35E-14	815	TGFBR1, ACVR1B, ACVR1C	RTK signaling

CMK	0.244218947	1.50E-06	379	RSK2	Other, kinases
BIBF-1120	0.242977347	4.07E-13	867	VEGFR, PDGFR, FGFR	RTK signaling
Sunitinib	0.242930807	1.76E-06	378	PDGFR, KIT, VEGFR, FLT3, RET, CSF1R	RTK signaling
(5Z)-7-Oxozeaenol	0.242325121	5.34E-13	863	TAK1	Other, kinases
GSK690693	0.242181872	6.05E-13	860	AKT1, AKT2, AKT3	PI3K/MTOR signaling
Apitolisib	0.238889115	1.42E-12	856	mTOR, PI3K	PI3K/MTOR signaling
AZD6738	0.238300264	7.69E-13	881	ATR	Genome integrity
AT-7519	0.237574198	2.20E-12	851	CDK1, CDK2, CDK4, CDK6, CDK9	Cell cycle
KU-55933	0.237511592	6.25E-13	894	ATM	Genome integrity
FGFR_3831	0.236929331	1.05E-12	881	FGFR1, FGFR2, FGFR3, FGFR4	RTK signaling
JQ1	0.236464664	4.69E-13	883	BRD2, BRD3, BRD4, BRDT	Chromatin other
Vinblastine	0.234158338	1.42E-12	892	Microtubule destabiliser	Mitosis
FR-180204	0.231780535	5.46E-12	863	ERK1, ERK2	ERK MAPK signaling
AZ20	0.231645167	2.70E-12	889	ATR	Genome integrity
MetAP2 Inhibitor, A832234	0.231631932	7.26E-12	854	MetAP2	Other
Wee1 Inhibitor	0.229189676	4.20E-11	809	WEE1, CHEK1	Cell cycle
PLK_6522	0.228301082	1.03E-11	867	PLK1, PLK2, PLK3	Cell cycle
TW 37	0.227309906	7.18E-12	888	BCL2, BCL-XL, MCL1	Apoptosis regulation
Pyrimethamine	0.226627582	7.92E-06	381	Dihydrofolate reductase (DHFR)	Other
PARP_0108	0.222936203	2.26E-11	880	PARP1, PARP2, PARP6	Genome integrity
AZD1208	0.221924104	2.58E-11	883	PIM1, PIM2, PIM3	Other, kinases
Omipalisib	0.219520652	6.89E-11	864	PI3K (class 1), MTORC1, MTORC2	PI3K/MTOR signaling
Phenformin	0.219168789	1.00E-10	852	Biguanide agent	Other
KIN001-042	0.216652687	1.14E-10	867	GSK3B	WNT signaling
Capivasertib	0.214093967	1.18E-10	887	AKT	PI3K/MTOR signaling
Kobe2602	0.212719393	3.09E-10	858	RAS effector	RTK signaling
Z-LLNle-CHO	0.208812048	4.19E-05	379	gamma-secretase	Other
Seliciclib	0.207304597	4.99E-05	377	CDK2, CDK7, CDK9	Cell cycle
QS11	0.20498394	2.88E-09	824	ARFGAP1	Other
NSC-87877	0.204694864	2.78E-09	828	SHP-1 (PTPN6), SHP-2 (PTPN11)	Other
HG-5-113-01	0.204422159	1.04E-05	458	LOK, LTK, TRCB, ABL(T315I)	Other
AZ628	0.203778271	5.76E-05	384	BRAF	ERK MAPK signaling
EHT-1864	0.203403252	9.14E-10	890	RAC1, RAC2, RAC3	Cytoskeleton
ZM447439	0.203090752	8.53E-10	896	AURKA, AURKB	Mitosis
Piperlongumine	0.202480626	1.54E-09	874	Induces reactive oxygen species	Other
Dactolisib	0.202172144	1.38E-09	882	PI3K (class 1), MTORC1, MTORC2	PI3K/MTOR signaling
Rucaparib	0.199250737	2.06E-09	889	PARP1, PARP2	Genome integrity
ZSTK474	0.193945034	9.82E-09	860	PI3K (class 1)	PI3K/MTOR signaling
DMOG	0.193165271	2.11E-08	828	HIF-PH	Metabolism
BPTES	0.192292099	8.93E-09	880	GLS	Other
AZD2014	0.190823854	1.12E-08	882	mTORC1, mTORC2	PI3K/MTOR signaling
AZD1480	0.190691958	1.12E-08	883	JAK1, JAK2	Other, kinases
Talazoparib	0.190337215	1.85E-08	860	PARP1, PARP2	Genome integrity
YK-4-279	0.189456195	1.72E-07	750	RNA helicase A	Other
PARP_9482	0.181984118	5.73E-08	877	PARP1, PARP2, PARP5a	Genome integrity
AICA Ribonucleotide	0.181964702	4.83E-08	887	AMPK agonist	Metabolism
AZD3514	0.181684107	5.72E-08	880	AR	Hormone-related
LFM-A13	0.181629455	1.52E-07	824	BTK	Other, kinases
LCL161	0.180859287	9.94E-08	856	XIAP, cIAP1, cIAP2	Apoptosis regulation
HG6-64-1	0.179183038	2.21E-07	825	BRAF	ERK MAPK signaling
FTY-720	0.175586438	2.19E-07	860	S1P	Other
Rapamycin	0.175201934	0.001159768	341	MTORC1	PI3K/MTOR signaling
Cisplatin	0.174103304	1.38E-07	822	DNA crosslinker	DNA replication
MCT4_1422	0.173626517	2.12E-07	882	MCT4	Other
Pilaralisib	0.172310662	3.26E-07	868	PI3K	PI3K/MTOR signaling
Dacinostat	0.17199193	6.92E-07	823	HDAC1	Chromatin histone acetylation
Etoposide	0.170505457	7.77E-07	830	TOP2	DNA replication
AZD6482	0.167947247	3.48E-07	810	PI3Kbeta	PI3K/MTOR signaling
Palbociclib	0.167221091	8.91E-07	854	CDK4, CDK6	Cell cycle
S-Trityl-L-cysteine	0.165956517	0.001202184	378	KIF11	Mitosis
PF-4708671	0.165140855	8.52E-07	879	S6K1	PI3K/MTOR signaling
JAK3_7406	0.165103421	8.21E-07	882	JAK3	Other, kinases
Shikonin	0.164363859	2.10E-06	824	not defined	Other
Pazopanib	0.161666904	3.32E-06	819	CSF1R, KIT, PDGFRA, PDGFRB	RTK signaling
CCT007093	0.161156665	1.42E-06	886	PPM1D	Cell cycle
Avagacestat	0.159527332	1.22E-06	819	Amyloid beta20, Amyloid beta40	Other
Entinostat	0.159520271	0.001890241	377	HDAC1, HDAC3	Chromatin histone acetylation
BAM7	0.159277174	2.71E-06	859	Bax activator	Other
VX-11e	0.156937802	3.61E-06	863	ERK2	ERK MAPK signaling
CGP-082996	0.155105883	0.002462106	379	CDK4	Cell cycle
Sorafenib	0.154627911	0.002506786	380	PDGFR, KIT, VEGFR, RAF	RTK signaling
LGK974	0.152903498	6.92E-06	857	PORCN	WNT signaling
Motesanib	0.152357177	4.86E-06	892	VEGFR, RET, KIT, PDGFR	RTK signaling
AZD4877	0.149721321	8.09E-06	881	EG5	Mitosis
GNF-2	0.149522604	0.003614999	377	BCR-ABL	ABL signaling
CAP-232, TT-232, TLN-232	0.147301196	1.49E-05	857	Glycolysis	Metabolism
QL-VIII-58	0.146347162	0.001648321	460	MTOR, ATR	Other
Cytarabine	0.145093665	1.47E-05	885	Antimetabolite	Other
AZD5582	0.144618668	1.83E-05	871	IAP	Other
WHI-P97	0.14339026	2.36E-05	863	JAK3	Other, kinases
Obatoclax Mesylate	0.142899245	4.19E-05	816	BCL2, BCL-XL, BCL-W, MCL1	Apoptosis regulation
CPI-613	0.142550132	2.72E-05	860	Metabo, Mitochondria	Other
JNK Inhibitor VIII	0.142516193	1.93E-05	892	JNK	JNK and p38 signaling
RAF_9304	0.142019556	2.30E-05	882	ARAF, BRAF, CRAF	ERK MAPK signaling
AZD8186	0.141966889	2.40E-05	879	PI3Kbeta, PI3Kdelta	PI3K/MTOR signaling
Enzastaurin	0.141832527	2.96E-05	861	PKCB	Other, kinases
rTRAIL	0.139338309	3.80E-05	868	TRAIL receptor agonist	Apoptosis regulation
Gemcitabine	0.136618254	3.48E-05	842	Pyrimidine antimetabolite	DNA replication

ICL1100013	0.136536536	5.78E-05	862	N-myristoyltransferase 1/2	Other
AKT inhibitor VIII	0.135878772	5.47E-05	845	AKT1, AKT2, AKT3	PI3K/MTOR signaling
Doxorubicin	0.135304328	4.10E-05	850	Anthracycline	DNA replication
Tozasertib	0.134027268	0.009458936	374	AURKA, AURKB, AURKC, others	Mitosis
GSK1904529A	0.133275958	0.000126905	822	IGF1R, IR	RTK signaling
JQ12	0.132712309	0.000129368	827	HDAC1, HDAC2	Chromatin histone acetylation
FTI-277	0.131229591	0.000148426	831	Farnesyl-transferase (FNTA)	Other
Bosutinib	0.130817821	8.75E-05	894	SRC, ABL, TEC	Other, kinases
Pevonedistat	0.129174965	0.000848947	664	NAE	Other
Serdemetan	0.127577075	0.000139085	887	MDM2	p53 pathway
AS605240	0.126214228	0.000210174	858	PI3Kgamma	PI3K/MTOR signaling
IGFR_3801	0.122213172	0.000281414	879	IGFR1	IGF1R signaling
Sepantronium bromide	0.120696259	0.000512402	825	BIRC5	Apoptosis regulation
BMS-509744	0.119486467	0.019811477	380	ITK	Other, kinases
OSU-03012	0.119260769	0.000611857	822	PDK1 (PDPK1)	Metabolism
JNK-9L	0.117794612	0.000672971	830	JNK2, JNK3	JNK and p38 signaling
CCT-018159	0.111672839	0.000881074	884	HSP90	Protein stability and degradation
TWS119	0.106093705	0.00174777	868	GSK3	WNT signaling
TTK_3146	0.105433227	0.001715559	882	TTK	Mitosis
Luminespib	0.10341465	0.003101269	816	HSP90	Protein stability and degradation
TGX221	0.099111563	0.053553531	380	PI3Kbeta	PI3K/MTOR signaling
Trichostatin A	0.096772048	0.004599492	856	HDAC	Chromatin histone acetylation
Midostaurin	0.095429612	0.005873491	832	PKC, PPK, FLT1, c-FGR, others	Other
PF-562271	0.095209079	0.006428693	818	FAK, FAK2	Cytoskeleton
Bexarotene	0.094257078	0.007086807	815	Retinioic X receptor (RXR) agonist	Other
Wnt-C59	0.0939608	0.005852048	859	PORCN	WNT signaling
AZD5438	0.090711025	0.007088621	880	CDK2	Cell cycle
MG-132	0.089955354	0.079889469	380	Proteasome, CAPN1	Protein stability and degradation
CGP-60474	0.08385211	0.103121415	379	CDK1,CDK2,CDK5,CDK7,CDK9, PKC	Cell cycle
GW843682X	0.081761273	0.11347113	376	PLK1	Cell cycle
Bryostatin 1	0.079335027	0.023007212	821	PKC	Other
Pelitinib	0.078937206	0.020384952	863	EGFR	EGFR signaling
RO-3306	0.078054325	0.019453513	896	CDK1	Cell cycle
Doramapimod	0.077567114	0.021379784	880	p38, JNK2	JNK and p38 signaling
HG-5-88-01	0.073261033	0.117828131	457	EGFR, ADCK4	Other, kinases
Embelin	0.071654738	0.03962369	825	XIAP	Apoptosis regulation
GSK650394	0.071512526	0.040998522	817	SGK2, SGK3	Other, kinases
Pictilisib	0.071349613	0.032239074	862	PI3K (class 1)	PI3K/MTOR signaling
BI-2536	0.068357256	0.185370243	377	PLK1, PLK2, PLK3	Cell cycle
KU-60019	0.067491051	0.044242813	889	ATM	Genome integrity
Vinorelbine	0.067293787	0.052481712	831	Microtubule destabiliser	Mitosis
PF-00299804	0.06633556	0.051816713	860	EGFR, ERBB2, ERBB4	RTK signaling
CP724714	0.065947212	0.052654022	864	ERBB2	RTK signaling
MK-2206	0.06557905	0.055404527	854	AKT1, AKT2	PI3K/MTOR signaling
Mitomycin-C	0.063004894	0.071007483	822	DNA crosslinker	DNA replication
Linsitinib	0.059180762	0.09054261	819	IGF1R	IGF1R signaling
IAP_7638	0.05444574	0.108334375	871	IAP	Other
AS601245	0.049073643	0.159820985	822	JNK1, JNK2, JNK2	JNK and p38 signaling
CI-1033	0.048902121	0.150471684	866	EGFR, ERBB2, ERBB4	RTK signaling
BMS-536924	0.045856086	0.170002851	625	IGF1R, IR	IGF1R signaling
Tipifarnib	0.041169613	0.238088522	823	Farnesyl-transferase (FNTA)	Other
Elesclomol	0.037383734	0.267407125	882	HSP90	Protein stability and degradation
Bleomycin	0.034776897	0.323489015	808	dsDNA break induction	DNA replication
A-83-01	0.034402976	0.314434861	857	TGFB	Other
FGFR_0939	0.033427449	0.321110989	883	FGFR4	RTK signaling
JW-7-52-1	0.033312264	0.525234424	366	MTOR	PI3K/MTOR signaling
Fulvestrant	0.029064277	0.388889648	881	ESR1	Hormone-related
PARP_9495	0.023333825	0.490863614	874	PARP1, PARP2, PARP7	Genome integrity
WH-4-023	0.019370168	0.708117139	376	SRC, LCK	Other, kinases
Saracatinib	0.01751774	0.731202694	387	ABL, SRC	Other, kinases
WZ-1-84	0.016116148	0.754484384	379	BMX	Other, kinases
Thapsigargin	0.011122201	0.752697657	805	SERCA	Other
Bortezomib	0.009672773	0.851902542	375	Proteasome	Protein stability and degradation
GW441756	0.005166958	0.877528604	892	NTRK1	RTK signaling
Bleomycin (50 uM)	0.003749928	0.911400003	883	dsDNA break induction	DNA replication
CI-1040	0.003455464	0.918424459	881	MEK1, MEK2	ERK MAPK signaling
BMS-754807	-0.000451691	0.98971475	817	IGF1R, IR	RTK signaling
Paclitaxel	-0.000468513	0.992765939	377	Microtubule stabiliser	Mitosis
A-770041	-0.000640343	0.99008656	379	LCK, FYN	Other, kinases
A-443654	-0.008405579	0.870438508	379	AKT1, AKT2, AKT3	PI3K/MTOR signaling
AZD1332	-0.009445925	0.779249433	883	NTRK1, NTRK2, NTRK3	RTK signaling
AZD8835	-0.009785219	0.772547828	875	PI3Kalpha, PI3Kdelta	PI3K/MTOR signaling
IAP_5620	-0.020246457	0.550228639	873	IAP	Other
NVP-TAE684	-0.020848098	0.683824376	384	ALK	RTK signaling
Bleomycin (10 uM)	-0.026402281	0.434072757	880	dsDNA break induction	DNA replication
XAV939	-0.04643786	0.171901053	867	TNKS1, TNKS2	WNT signaling
PI3Ka_4409	-0.047121035	0.163246553	877	PI3Kalpha, PI3Kdelta	PI3K/MTOR signaling
Epothilone B	-0.055684751	0.111076636	820	Microtubule stabiliser	Mitosis
Gefitinib	-0.065177974	0.052186891	888	EGFR	EGFR signaling
Dasatinib	-0.066139382	0.201888031	374	ABL, SRC, Ephrins, PDGFR, KIT	RTK signaling
JNJ38877605	-0.06894761	0.042512756	866	MET	RTK signaling
Erlotinib	-0.070708054	0.17124073	376	EGFR	EGFR signaling
FH535	-0.07378659	0.034636429	820	PPARgamma, PPARdelta	Other
TANK_1366	-0.077774368	0.020887879	882	Tankyrase 1/2 (PARP5a, PARP5b)	Genome integrity
AST-1306	-0.08183433	0.016757865	854	EGFR, ERBB4	RTK signaling
Selumetinib	-0.107655442	0.001225932	856	MEK1, MEK2	ERK MAPK signaling
Docetaxel	-0.113273124	0.000691229	894	Microtubule stabiliser	Mitosis
Dyrk1b_0191	-0.120112171	0.000350173	882	DYRK1B	Other, kinases

Cetuximab	-0.153633065	8.75E-06	830	EGFR	EGFR signaling
Lapatinib	-0.164801838	0.001302104	378	EGFR, ERBB2	RTK signaling
Refametinib	-0.181890451	3.84E-08	854	MEK1, MEK2	ERK MAPK signaling
EphB4_9721	-0.206368767	6.56E-10	879	EPHB4	RTK signaling
PD0325901	-0.214373008	1.26E-10	882	MEK1, MEK2	ERK MAPK signaling
Trametinib	-0.222496765	6.64E-11	842	MEK1, MEK2	ERK MAPK signaling
Tanespimycin	-0.276020916	0	894	HSP90	Protein stability and degradation
AZD8931	-0.290702503	0	879	EGFR, ERBB2, ERBB3	RTK signaling
Afatinib	-0.3133726	0	892	ERBB2, EGFR	EGFR signaling
