# Supplementary material for: Characterization of the Estrogen Response Helps to Predict Prognosis and Identify Potential Therapeutic Targets in Cholangiocarcinoma
Source: Front Oncol. 2022 May 19;12:870840. doi: 10.3389/fonc.2022.870840 (PMC9162778; doi:10.3389/fonc.2022.870840)
Supplement: Supplementary file 2 [file DataSheet_2.pdf]

Supplementary figure 1. Overview of the study

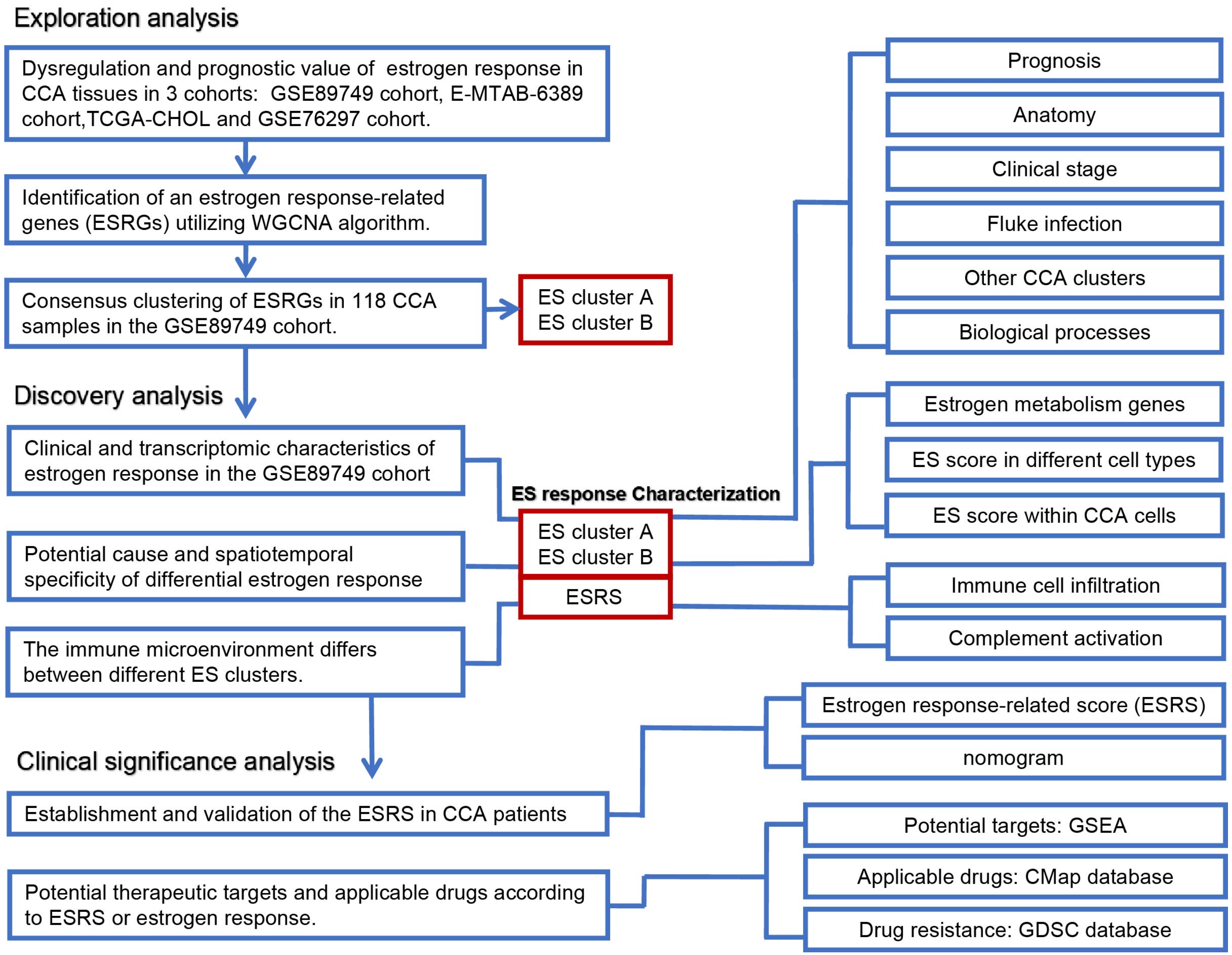

Supplementary figure 2. Estrogen response predicts poor prognosis in CHOL and differs among different cancer types.

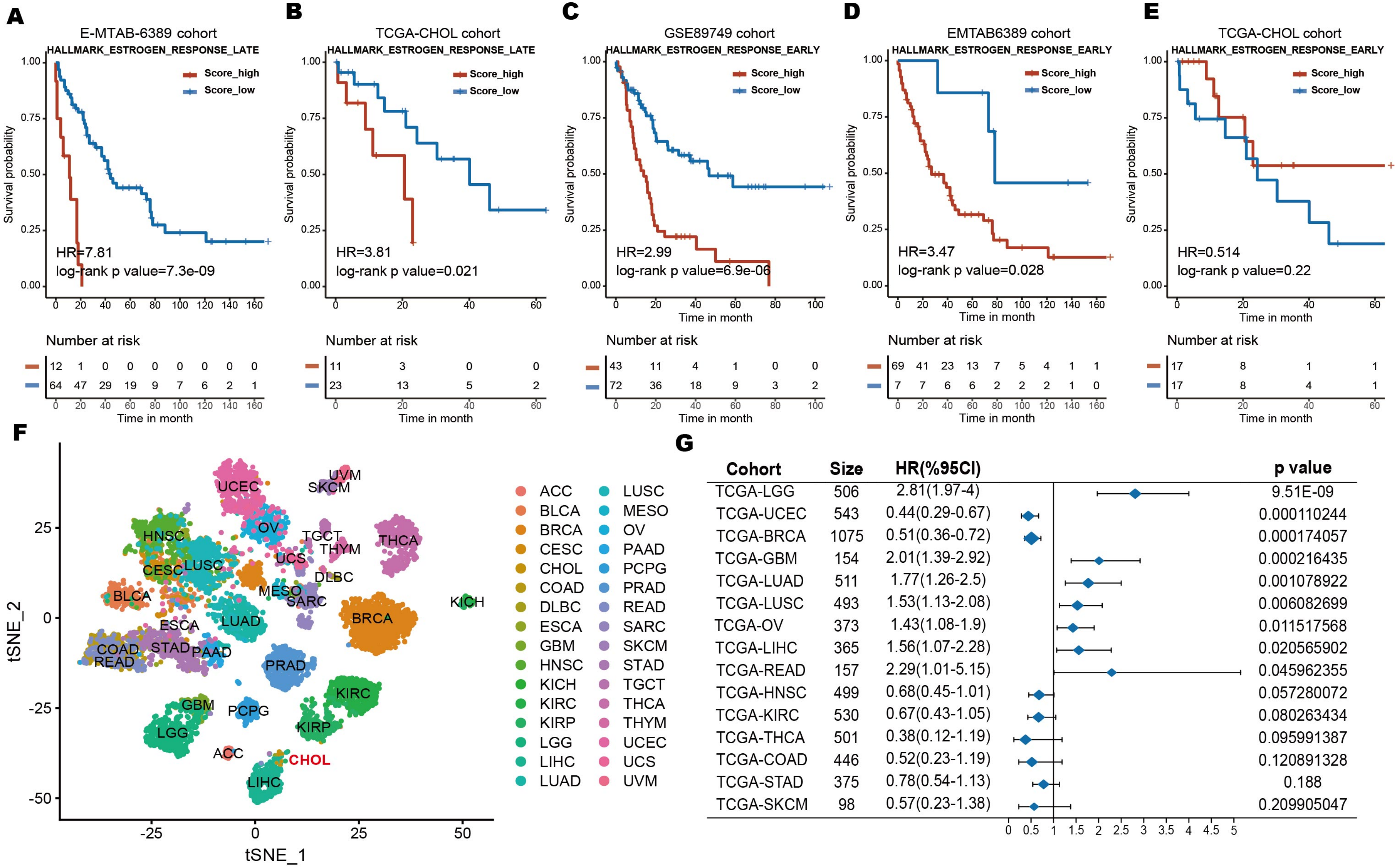

A-**E**, Kaplan–Meier curves for overall survival of the patients with low or high GSVA score of "HALLMARK_ESTROGEN_RESPONSE_LATE" or "HALLMARK_ESTROGEN_RESPONSE_EARLY" in the GSE89749 cohort, E-MTAB-6389 cohort or TCGA-CHOL cohort. **F**, TSNE plot according to the expression of genes in "HALLMARK_ESTROGEN_RESPONSE_LATE" for all the 32 types of cancerous tissues in TCGA cohort. **G**, Forest plot showing the prognostic value of GSVA score of "HALLMARK_ESTROGEN_RESPONSE_LATE" in 15 types of solid tumor.

Supplementary figure 3. Clinical and transcriptomic characteristics of ES clusters in GSE89749 cohort.

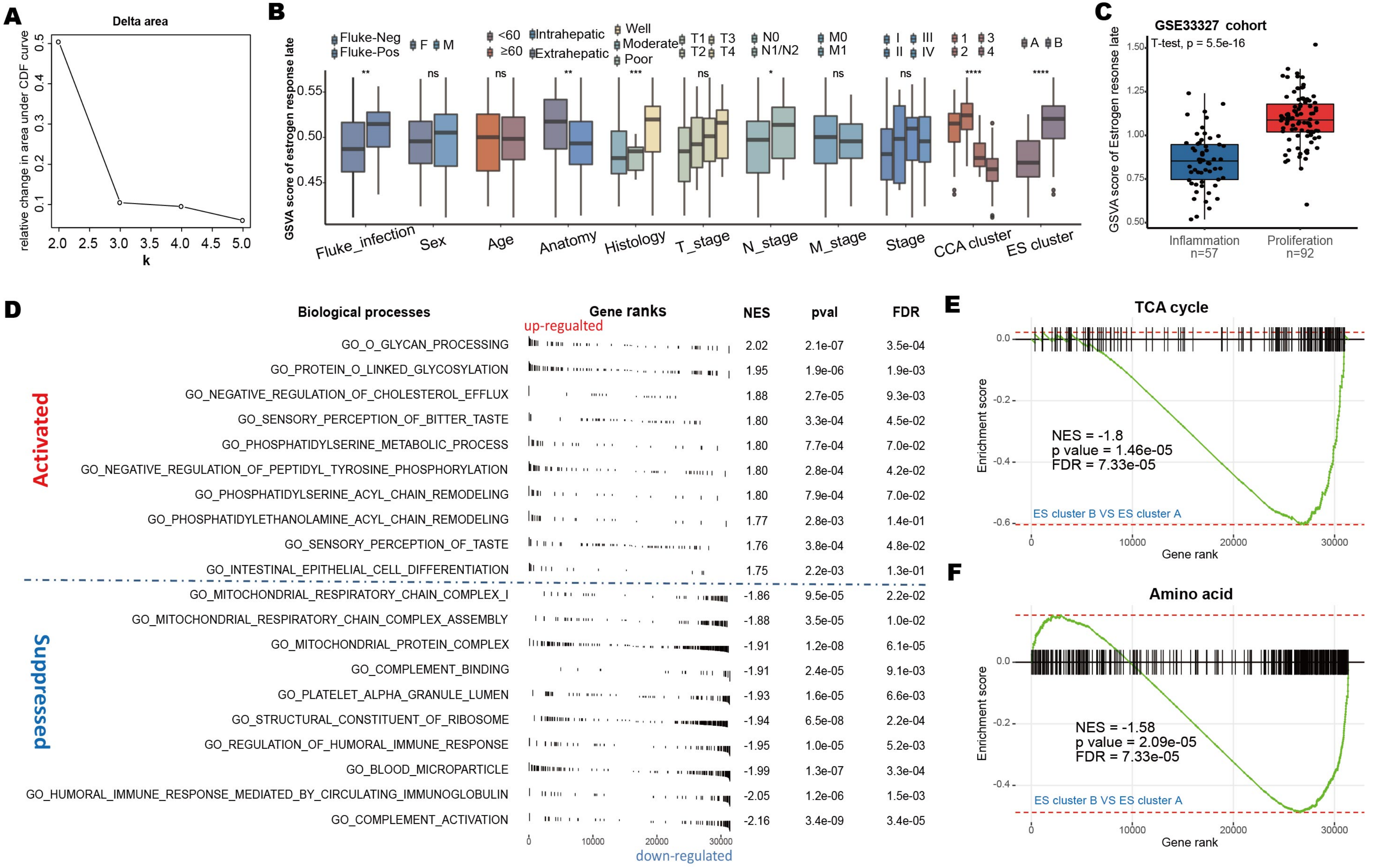

A, Optical clustering chosen by delta area. **B**, Boxplot showing the GSVA score of “HALLMARK_ESTROGEN_RESPONSE_LATE” in different fluke infection status, sex, age, anatomy, histology, T stage, N stage, M stage, clinical stage, CCA cluster and ES cluster. C, Boxplot showing the difference of estrogen response in inflammation and proliferation subtypes in GSE33327 cohort. **D**, The top biological processes activated or suppressed in ES cluster B versus ES cluster A generated by GSEA. **E** and **F**, GSEA plots exhibit the enrichment of metabolism of TCA cycle and amino acid in ES cluster B.

Supplementary figure 4. Estrogen response and the expression of KRT19, ANXA4, COMT, HSD17B1 in single cell level.

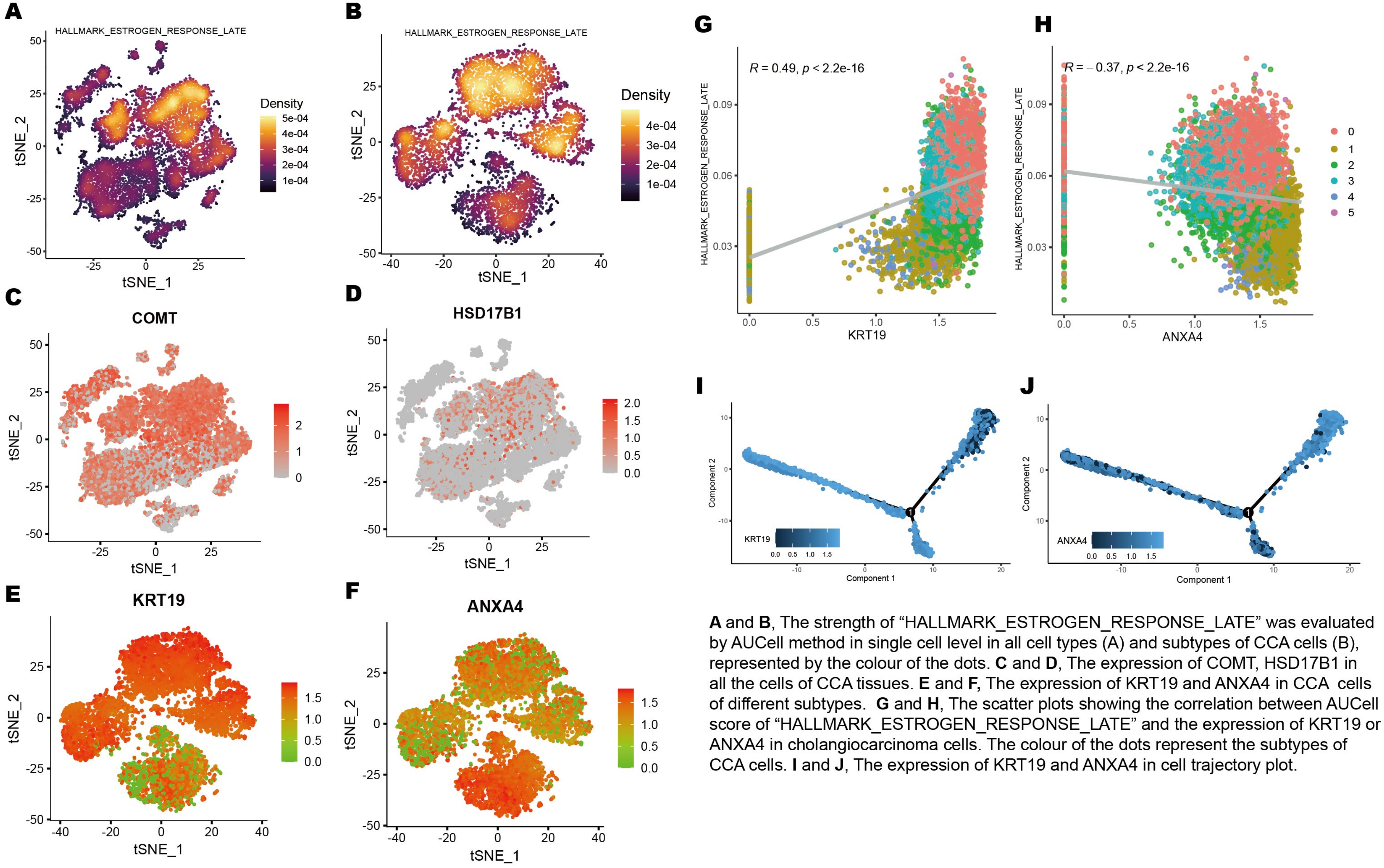

A and **B**, The strength of "HALLMARK_ESTROGEN_RESPONSE_LATE" was evaluated by AUCell method in single cell level in all cell types (A) and subtypes of CCA cells (B), represented by the colour of the dots. **C** and **D**, The expression of COMT, HSD17B1 in all the cells of CCA tissues. **E** and **F,** The expression of KRT19 and ANXA4 in CCA cells of different subtypes. **G** and **H**, The scatter plots showing the correlation between AUCell score of "HALLMARK_ESTROGEN_RESPONSE_LATE" and the expression of KRT19 or ANXA4 in cholangiocarcinoma cells. The colour of the dots represent the subtypes of CCA cells. **I** and **J**, The expression of KRT19 and ANXA4 in cell trajectory plot.

Supplementary figure 5. Immune cell infiltration and complement activation status in cholangocarcinoma.

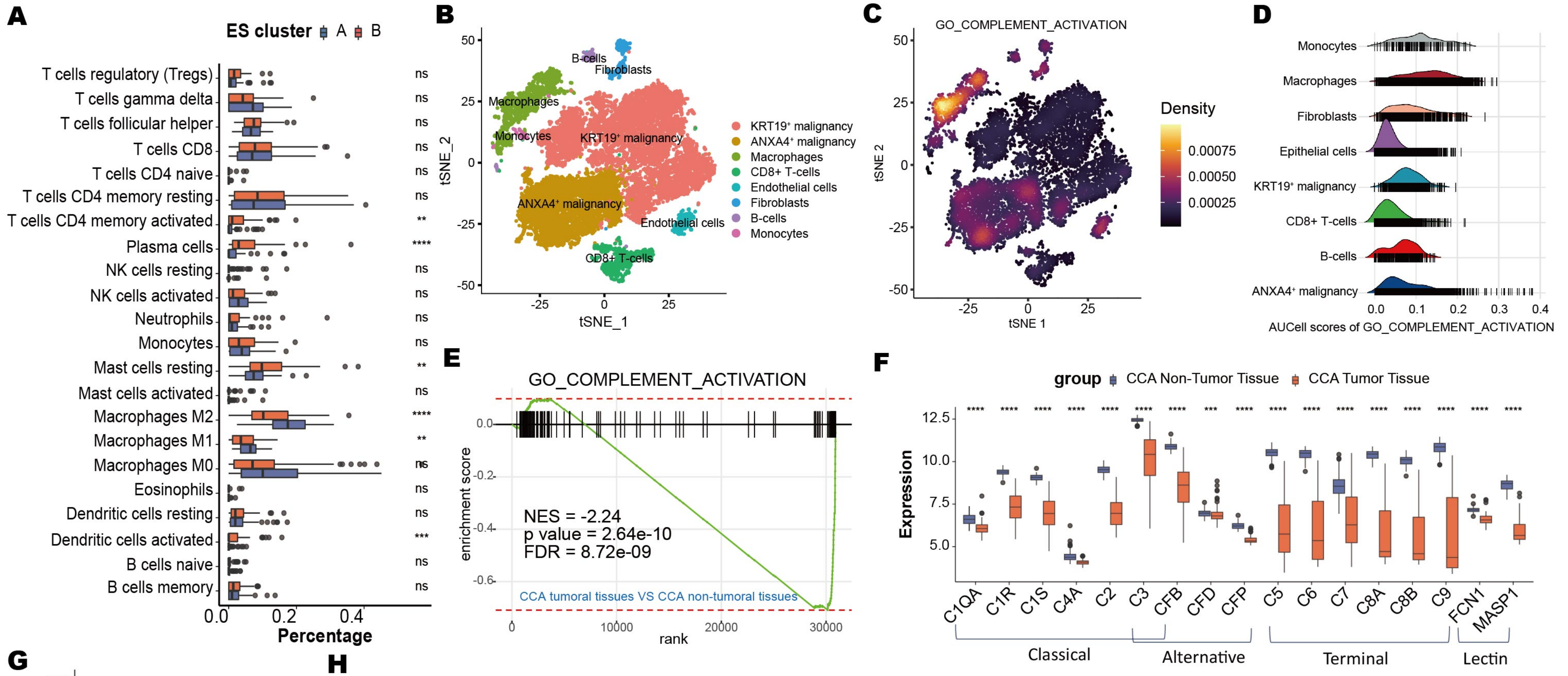

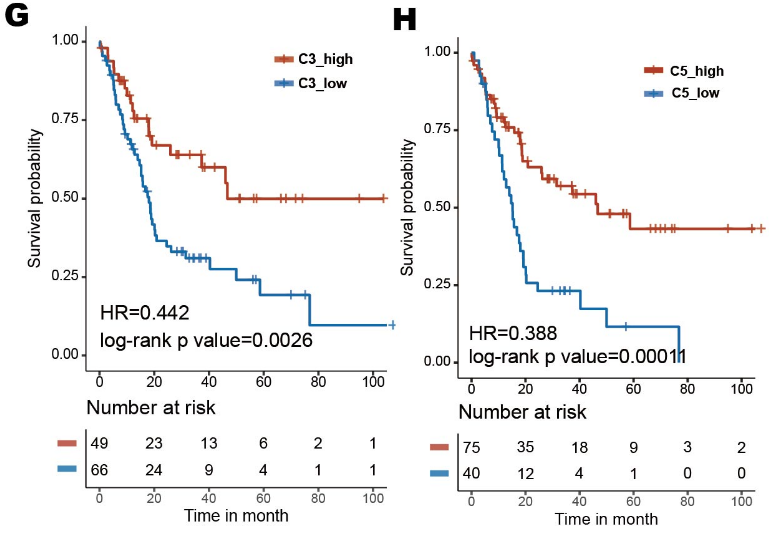

A, Differential infiltration of immune cells in ES cluster A and B in GSE89749 cohort estimated by cibersort. **B**, Annotation of single cell types in 5 CCA tissues. The cholangiocarcinoma cells were further divided into KRT19⁺ and ANXA4⁺ malignancy according to the expression or KRT19 and ANXA4. **C**, The strength of “GO_COMPLEMENT_ACTIVATION” was evaluated by AUCell method and exhibited in the TSNE plot by colour. **D**, Ridge plot showing the AUCell score of “GO_COMPLEMENT_ACTIVATION” in all cell types in different cell types of CA tissues. **E**, The GSEA enrichment plot showing the enrichment of “GO_COMPLEMENT_aCTIVATION” in CCA tumor tissues compared with CCA non-tumor tissues. **F**, Boxplot showing the expression of complement gene expression in CCA tumor tissues and CCA non-tumor tissues. **G**- **H**, Kaplan–Meier curves for overall survival of the patients with low or high expression of C3 and C5 in the GSE89749 cohort.

Supplementary figure 6. Potential therapeutic targets and drugs should not be adopted according to ES response.

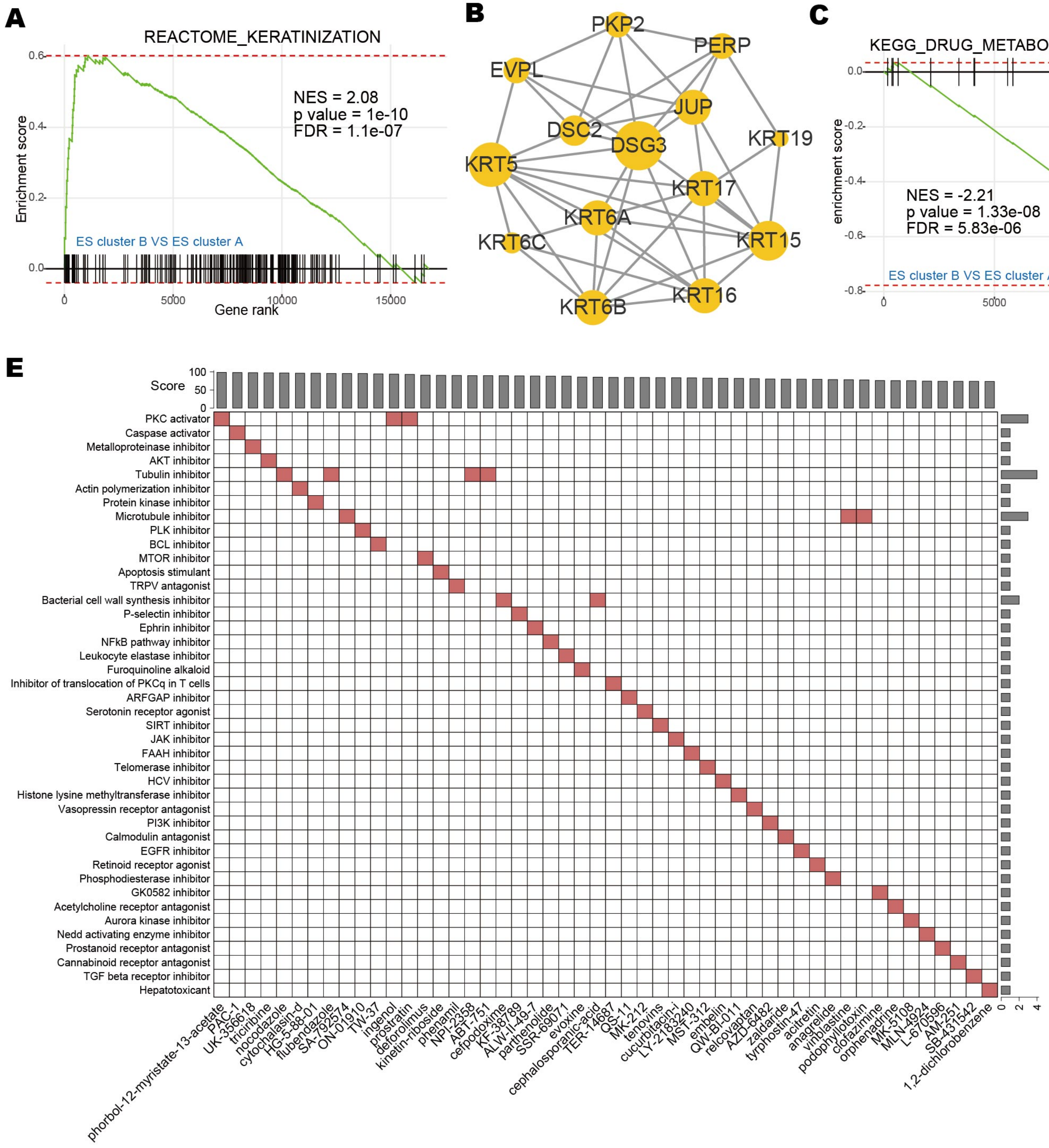

A and **C**, GSEA indicated that “REACTOME_KERATINIZATION” was top enriched pathway in ESRS_high group and “KEGG_DRUG_METABOLISM_CYTOCHROME_P450” was top enriched pathway in ESRS_low group. **B** and **D**, Two protein interaction networks were generated out of differential genes in “REACTOME_KERATINIZATION” (B) and “KEGG_DRUG_METABOLISM_CYTOCHROME_P450” (D). The size of the dot represents the fold change of the genes between ESRS_high and ESRS_low groups. **E** The differetially espressed genes were submitted to CMap mode-of-action (MoA) analysis and the results showed 42 mechanisms of action shared by top 50 compounds which show similar impact on cancer cells to ESRS which should be dismissed for ESRS_high patients.
